# Strontium isotope and trajectory method elucidating overseas migration of *Mythimna separata* to Japan

**DOI:** 10.1016/j.isci.2024.111160

**Published:** 2024-10-11

**Authors:** Naoya Hidaka, Caihong Tian, Shengnan Zhang, Gaku Akiduki, Guoping Li, Ichiro Tayasu, Ki-Cheol Shin, Tokumitsu Niiyama, Gao Hu, Shimin Li, Akira Otuka, Hongqiang Feng

**Affiliations:** 1 Institute for Plant Protection, National Agriculture and Food Research Organization, Koshi, Kumamoto 861-1192, Japan; 2 International Joint Research Laboratory for Crop Protection of Henan, Institute of Plant Protection, Henan Academy of Agricultural Sciences, Zhengzhou, Henan 450002, P.R. China; 3 Research Institute for Humanity and Nature, Kyoto 603-8047, Japan; 4 Akita Plant Protection Station, Akita 010-1231, Japan; 5 College of Plant Protection, Nanjing Agricultural University, Nanjing, Jiangsu 210095, P.R. China; 6 Institute of Plant Protection, Luohe Academy of Agricultural Sciences, Luohe, Henan 462300, P.R. China

**Keywords:** Ecology, Entomology, Evolutionary biology

## Abstract

The oriental armyworm, *Mythimna separata*, generally migrates from eastern to northeastern China in early summer, and some individuals are believed to migrate overseas to Japan depending on meteorological conditions. This potential migration was investigated with the immigrants’ strontium radiogenic isotope ratio ^87^Sr/^86^Sr and backward flight trajectories from Japanese trapping sites. The results showed that the ^87^Sr/^86^Sr ratios of Chinese reared *M. separata* were significantly higher than those of reared insects of Japanese immigration areas. As some individuals trapped in western Japan had ^87^Sr/^86^Sr ratios higher than a statistical discriminating ratio, they likely originated in China. Trajectory analysis also indicated those individuals might have originated from the East Asian continent, such as the first-generation outbreak region in China and their migration waypoint regions. Our analysis, thus, suggests direct or multistep overseas migration of individual *M. separata* from the East Asian continent to Japan, giving insight into migration pathways and population dynamics.

## Highlights

• Oriental armyworms form a trap catch peak in early summer in western Japan
• Sr isotope ratios of the trapped moths show high values of the Chinese natal origin
• Backward trajectories from the trap site reach over the seasonal source in China
• Sr isotope and trajectory method elucidate overseas migration from China to Japan

## Graphical abstract

**Figure:**
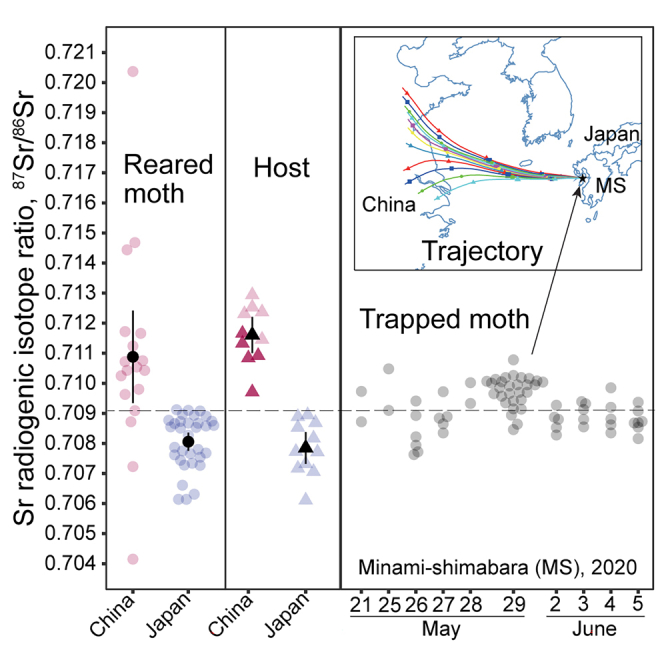

## Introduction

The migration of organisms is generally a seasonal movement over relatively long distances from a habitat where the organism’s survival conditions are deteriorating to a habitat where they are improving.^1^ The migration affects the range shift, mixing of the gene pool, community structure, and species interactions at the destination.^2^^,^^3^ Among migratory organisms, many migratory insects conduct seasonal migrations at the population level, resulting in large biomass inflows.^4^ Long-distance insect migrations across hundreds of kilometers have been widely observed in various locations on the globe, with some migratory insects constraining their movements to individual continents and others moving across oceans or seas. Migration and/or long-distance dispersal to remote islands has long been of interest to island ecologists, particularly with respect to the mode of movement to the islands (e.g., via wind, oceanic drift, or other dispersal vectors), meteorological processes, and the similarity in insect fauna between the island and the continent of origin.^3^ In addition, migration and economic ecologists investigate the associated behaviors and mechanism of overseas migration so as to achieve effective pest management.^5^

This study focuses on overseas migration of the oriental armyworm *Mythimna separata* Walker (Lepidoptera: Noctuidae), a devastating and long-distance migratory insect pest species.^6^^,^^7^ Since *M. separata* larvae cannot tolerate temperatures below 0°C–4°C, they overwinter in southern areas below 33°N in China and south-western Japan.^6^^,^^7^^,^^8^^,^^9^ The overwintering generation of *M. separata* adults in southern China <33°N migrates northwards from March to mid-April to the first-generation outbreak region between 32°N and 36°N in the Chinese Central Plains,^7^ where its favorite host plant, winter wheat, *Triticum aestivum*, is widely cultivated. Adults of the first generation emerge in late May to early June from this region, mainly migrating to northern and northeastern China, the Korean Peninsula, and potentially to northern or western Japan (the Aomori and Akita prefectures, Hokkaido and Kyushu district).^6^^,^^7^^,^^10^ This period will be referred to as early summer hereinafter.

**Figure 1:**
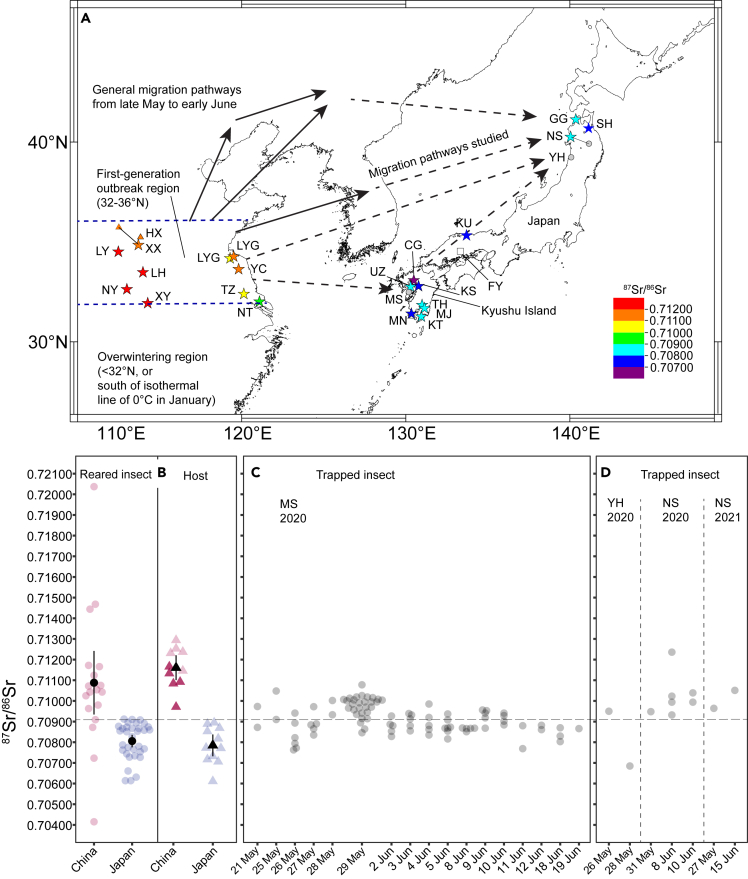
Strontium radiogenic isotope ratios (^87^Sr/^86^Sr) of the reference in Chinese emigration and Japanese immigration areas and *M. separata* caught in Japanese immigration area (A) Location map of the study area. The solid stars and gray solid circles in Japan indicate reference and trap sites, respectively (Tables S1 and S2). The solid stars and solid triangles in China indicate reference and previous wheat-grain collection sites, respectively (Table S1). The color of the stars indicates the ^87^Sr/^86^Sr ratio of the reference hosts. Two- or three-letter codes indicate the names of the cities and towns where host and insect samples were collected: GG, Goshogawara; SH, Shichinohe in Aomori prefecture; NS, Noshiro; YH, Yorihonjo in Akita prefecture; KU, Kotoura in Tottori prefecture; CG, Chikugo in Fukuoka prefecture; KS, Koshi in Kumamoto prefecture; UZ, Unzen; MS, Minami-Shimabara in Nagasaki prefecture; TH, Takaharu; MJ, Miyakonojo in Miyazaki prefecture; MN, Minami-Satsuma; and KT, Kimotsuki in Kagoshima prefecture, Japan; LYG, Lianyungang; YC, Yancheng; TZ, Taizhou; NT, Nantong in Jiangsu province; HX, Huixian; XX, Xinxiang; LY, Luoyang; LH, Luohe; NY, Nanyang; and XY, Xinyang in Henan province, China. The open square in western Japan indicates a previous monitoring site at Fukuyama (FY), Hiroshima prefecture for discussion. (B) Strontium radiogenic isotope ratios (^87^Sr/^86^Sr) of the reared *M. separata* (*red and blue solid circle*) and the host leaves (*red and blue solid triangle*) in Japan and China, respectively. The dark and light red triangles indicate the ^87^Sr/^86^Sr ratios of hosts from Jiangsu and Henan provinces, respectively. The black solid circles and triangles indicate average, and the black vertical lines standard deviation. (C) ^87^Sr/^86^Sr ratios of *M. separata* trapped in Nagasaki prefecture in 2020. (D) ^87^Sr/^86^Sr ratios of *M. separata* trapped in Akita prefecture in 2020 and 2021. The horizontal dashed lines in panels (B–D) indicate the cut-off ^87^Sr/^86^Sr ratio obtained by ROC analysis of the logistic curve (Figure 2).

Although the windborne long-distance migration of *M. separata* has been well studied in China using various methods, including a mark-release-and-recapture experiment, ground-based light trapping, and entomological radar, trajectory, and genetic analyses,^11^^,^^12^^,^^13^^,^^14^^,^^15^ overseas migration from China to Japan has not been studied in detail. Migrations of *M. separata* have been associated with severe outbreaks of larvae in late June to early July in northern Japan, where this species could not overwinter.^6^ The potential migration pathway of this species from eastern China to northern Japan has been depicted by capture of adults on ships in the Bohai Sea and Yellow Sea,^16^ and by simply referring to surface weather maps.^6^^,^^17^ Abrupt outbreaks of the moth occasionally occur in western Japan as well, suggesting potential migrations.^6^ However, these migration pathways remain far from clear. At the same time, it is expected that there also exists a Japanese domestic migration pathway of the first generation from western to northern regions.^9^ Although *M. separata* is able to overwinter in western Japan (Kagoshima to Hiroshima prefectures: FY in Figure 1A), the overwintering density is very low, e.g., <1 larva/m^2^ in Hiroshima, 1–2 larvae/m^2^ in Kagoshima.^9^ Thus, it remains unclear whether such a domestic migration pathway is relevant. The purpose of this study is to uncover possible migration pathways of *M. separata* immigrating into Japan by means of a widely used trajectory analysis^14^ and Strontium (Sr) isotope analysis.

Environmental isotopes reflecting abiotic and biotic environmental information in the bodies of individual organisms have also been utilized successfully to trace animal movement in the last two decades,^18^^,^^19^^,^^20^ and the application of such isotopes in insects has been reviewed by Quinby et al.^21^ The hydrogen stable isotope ratio *δ*^2^H of precipitation and surface water over a continent generally shows a systematic decrease with increasing latitude or elevation.^22^ The *δ*^2^H value of surface water on a continent exerts a strong influence on the *δ*^2^H value of insect herbivores through host plants.^22^ Therefore, this ratio is suitable for tracking south-northward migrations of an insect between winter and summer habitats.^18^ However, hydrogen is not very applicable to an eastward migration between latitudes that are not highly different, because the hydrogen stable isotope ratio does not change much within narrow latitudinal zones.

The strontium (Sr) radiogenic stable isotope ratio ^87^Sr/^86^Sr is an alternative tracer candidate for east–west migration.^23^ The Sr isotope ratio displays a unique and predictable pattern of variability on the Earth’s surface that follows the geological age and lithology of bedrocks in general.^23^^,^^24^ As rocks interact with the hydrosphere, atmosphere and biosphere, bedrock Sr is transferred to other reservoirs, such as soils, soil water and plants.^24^ Insect herbivores then take Sr from host plants, and there is no or little Sr isotope fractionation from the soil or plants to herbivores.^24^ Therefore, the ^87^Sr/^86^Sr ratio is a good tool for tracking animal migration.^20^ Note that while geological properties are the dominating influence on bioavailable Sr isotopes, other factors, such as human modification through an activity like agricultural practice, rain, and distance to shore (sea spray), have been shown to play a role in some regions.^25^^,^^26^^,^^27^^,^^28^ Also, bedrock Sr isotope signatures do not translate 1 to 1 to biospheric signatures, as different minerals have different Sr concentrations, isotopes signatures and weathering rates.^23^^,^^24^ Especially, factors modifying the ^87^Sr/^86^Sr ratio in agricultural fields are discussed later.

As examples of the migration study with the isotopic tracer, the ^87^Sr/^86^Sr ratio of the cotton bollworm, *Helicoverpa armigera* (Lepidoptera: Noctuidae), collected at several sites in Australia and New Zealand was divided into three groups, basically due to differences in the geological age of the soils.^29^ Possible sources of the spring northward migration of the monarch butterfly, *Danaus plexippus* Linnaeus, were assigned to narrow areas by comparing the ^87^Sr/^86^Sr and *δ*^2^H values of the wings of immigrants with dual fine isoscapes of these elements over the US,^30^ indicating the improved capability of plural isoscapes as a tool for tracing insect migrations. Wide areas of the Japanese islands are formed of volcanic bedrocks of a recent geological age, i.e., the Cenozoic Era, while the East Asian continent is mostly made of Mesozoic and Paleozoic bedrocks, which are older than some parts of the Japanese islands.^31^ The strontium of these bedrocks affects the strontium in the soils above them. Hence, the Sr radiogenic isotope ratio of insects and its host plants should differ between China and Japan,^24^ and this ratio could be used to infer the origin of overseas migrant insects.

However, trajectory analysis, which is commonly used by migration ecologists, has not frequently been employed in isotopic migration study, with the exception of a few studies on latitudinal migration using hydrogen isotopes.^32^^,^^33^ The migration of a windborne insect, especially an overseas long-distance migration, is a dynamical phenomenon occurring within limited temporal and spatial ranges. The insect in migration makes the best use of winds.^1^^,^^4^^,^^13^ In the case of nocturnal moths, it is believed that individuals mostly take off at dusk and fly primarily in a downwind direction over a limited flight duration to arrive at a destination area in each migration flight.^14^^,^^34^^,^^35^ Thus, immigrants trapped just after their arrival can mostly be traced backward by flight trajectories from a trapping site, thereby estimating their migration source and pathway.^14^^,^^34^^,^^35^ Trajectory analysis is one of the essential tools for migration study, but by itself it can present only circumstantial evidence of a migration. In contrast, isotopic analysis provides direct evidence. Therefore, the interdisciplinary combination of isotopic and trajectory analyses could improve the analysis of insect migration of the windborne type.

In order to present clear evidence of the migration of *M. separata* from China to Japan, we first measured the ^87^Sr/^86^Sr ratios of host plants collected in China and Japan and of *M. separata* adults raised on those host plants as a reference, and then identified overseas migration events based on the ^87^Sr/^86^Sr ratio of adults trapped in northern and western Japan, the established reference values, and the detailed source of the migrants as determined by backward trajectory analysis.

## Results

### Strontium isotope ratio of the reference

Reference host plants were maize for feed, *Zea mays* L., wheat, *Triticum aestivum* L., and orchard grass, *Dactylis glomerata* L. The ^87^Sr/^86^Sr ratio of the host leaves collected at major immigration sites in the Kyushu district (CG, UZ, KS, TH, MJ, MN), Chugoku district (KU) and Tohoku district (GC, SH, NH) in Japan ranged from 0.70611 to 0.70895 (0.70785 ± 0.00089) (Figures 1A and 1B). The ^87^Sr/^86^Sr ratio of the *M. separata* reared at the immigration sites ranged from 0.70614 to 0.70912 (0.70806 ± 0.00088) (Figure 1B). Linearity was observed between the ^87^Sr/^86^Sr ratios for the reared insects and those for the diet plants (linear regression *lm*, R^2^ = 0.908 and *y* = 0.9592*x* + 0.0289, Figure S1 in the supplemental information). Overall, the ^87^Sr/^86^Sr ratio of the Japanese references at the immigration sites ranged from 0.70611 to 0.70912 (0.70800 ± 0.00088).

The ^87^Sr/^86^Sr ratio of wheat leaves from Jiangsu province ranged from 0.70971 to 0.71166 (0.71089 ± 0.00074) and the ^87^Sr/^86^Sr ratio of wheat leaves from Henan province ranged from 0.71146 to 0.71294 (0.71232 ± 0.00054), and average and standard deviation of the ^87^Sr/^86^Sr ratio of all the Chinese wheat leaves are 0.71161 ± 0.00097 (Figure 1B). The Chinese host range appeared to be greater than those for the host plants and the reared insects from the Japan immigration areas, without any overlap in range (Figure 1B). The medians of the ^87^Sr/^86^Sr ratios of the host plants of the two Chinese provinces were significantly different (Brunner-Munzel test, Statistic = 8.1317, df = 8, *p*-value <0.01). The Sr isotope ratio of the Chinese reared insects ranged from 0.70415 to 0.72037 (0.71088 ± 0.00333).

**Figure 2:**
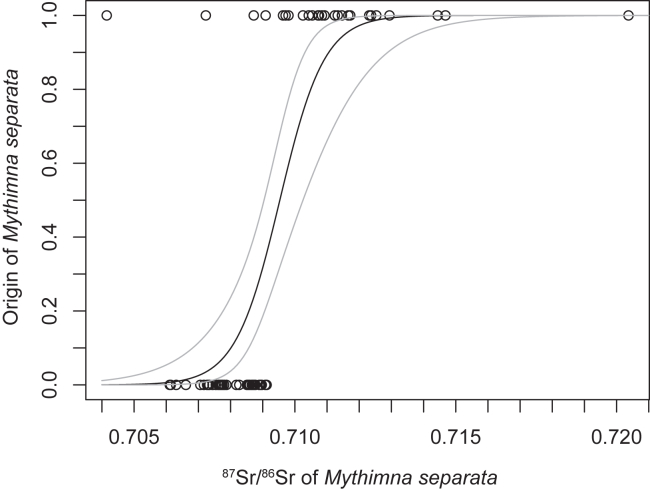
Logistic curve indicating the association between the origin of *Mythimna separata* and its ^87^Sr/^86^Sr ratio Gray lines show 95% confidence intervals. The origins considered here are Chinese origin or origin from the Japanese immigration sites investigated in this study. The ^87^Sr/^86^Sr ratio cut-off, 0.70909, for natal origin prediction was determined by ROC analysis of the curve (see the main text).

Overall, the medians of the ^87^Sr/^86^Sr ratio of the Chinese and Japanese references (the hosts and insects) were significantly different (Brunner-Munzel test, Statistic = 3.1375, df = 17.683, *p*-value <0.01). Logistic regression analysis indicated that the origin of *M. separata* was associated with the ^87^Sr/^86^Sr ratio of the insect (*p*-value <0.01). A predictive logistic curve (or produced model) with 95% confident intervals is given in Figure 2. ROC (receiver operating characteristic) analysis of the produced model indicated that the AUC (area under the ROC curve) was 0.924 (95% confidence intervals: 0.833–1.0), indicating proper predictability. The sensitivity, specificity, positive predictive value, and negative predictive value at the cut-off ^87^Sr/^86^Sr ratio of 0.70909, which was determined with the “closest.topleft” method, were 89.3%, 95.5%, 92.6%, and 93.3%, respectively. Note that although we simply use the cut-off value in the results section as a *tentative* value to discriminate Chinese origin and Japanese immigration area’s origin, there may be an overlap of the ^87^Sr/^86^Sr ratios of the Chinese and Japanese references. Refer to the discussion section.

### ^87^Sr/^86^Sr ratios of *M. separata* trapped in the Japanese immigration sites

At MS of Nagasaki prefecture, the ^87^Sr/^86^Sr ratios of 46 *M separata* insects were greater than the cut-off ^87^Sr/^86^Sr ratio (0.70909) (Figure 1C). Another 43 insects with Sr isotope ratios less than the cut-off, suggesting a probable origin of the local trapping area, were also found throughout the investigation period (Figure 1C).

At NS (*n* = 9) and YH (*n* = 2) of Akita prefecture, the results showed that all ^87^Sr/^86^Sr ratios of trapped *M. separata* were greater than the cut-off (Figure 1D) except for one insect with a very low ^87^Sr/^86^Sr ratio. which seemed likely to be an immigrant arriving from somewhere in Japan until 28 May 2020 at YH.

### Trajectory analysis

**Figure 3:**
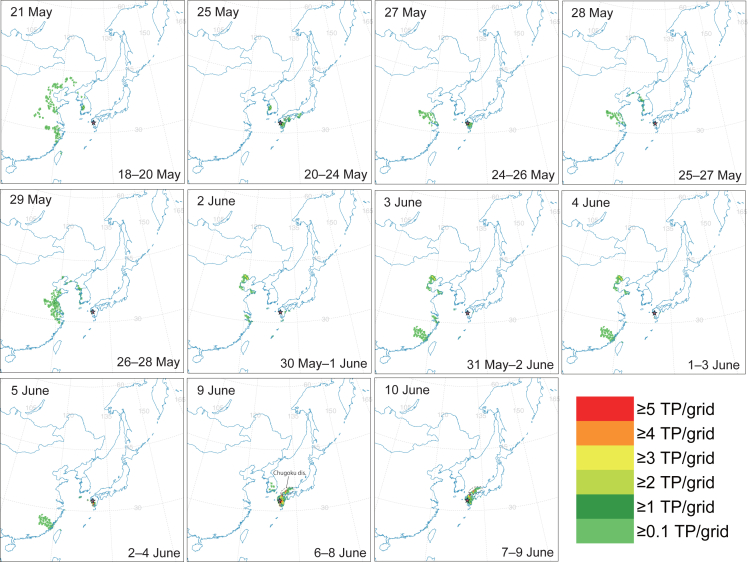
Frequency map of the terminal points of backward trajectories from Minami-Shimabara (MS) in 2020 Flight duration ranges from 13 h to 36 h. A color grid indicates the number of terminal points in the grid (TP/grid; grid size of 0.25 × 0.25°). A grid smaller than 1 TP/grid was produced by a smoothing function around neighboring grids. The date of sample collection is shown in the upper line of each map, and the analytical period (UTC) in the lower line. These dates of sample collection indicate insect catches with ^87^Sr/^86^Sr ratios above the cut-off ^87^Sr/^86^Sr ratio of 0.70909 obtained by ROC analysis of the logistic curve (Figure 2).

**Figure 4:**
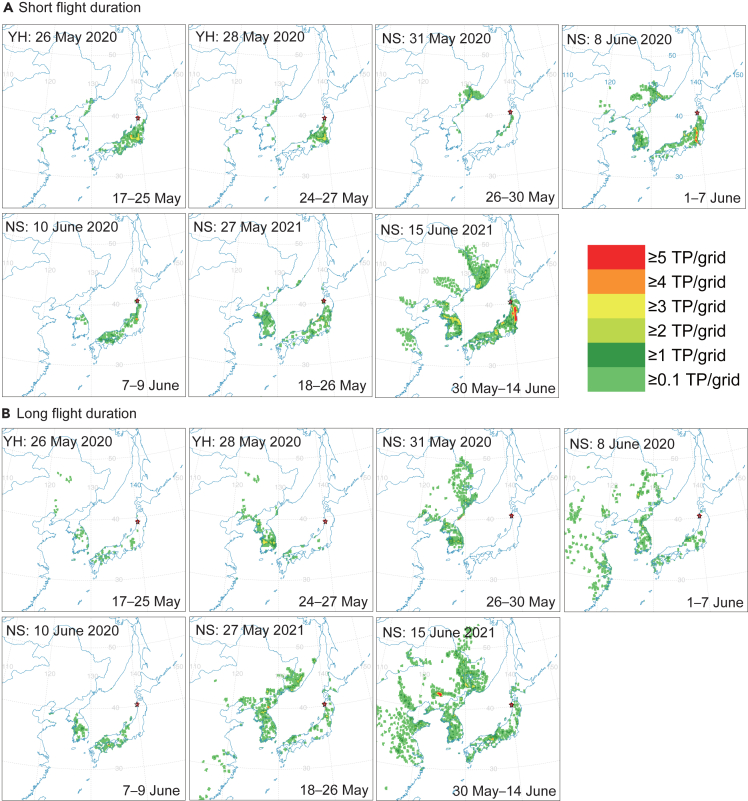
Frequency map of the terminal point of backward trajectories from northern Japan in 2020 and 2021 (A) Short flight duration (min 13 h, max 36 h) (B) Long flight duration (min 37 h, max 60 h). The starting height was between 0 and 1500 m. Analytical periods of the trajectory analysis for each date of sample collection are summarized in Table S7. The locality and date of sample collection are shown in the upper line, and the analytical period (UTC) is shown in the lower line in each map.

Backward trajectories from Nagasaki and Akita prefectures in analytical periods when individuals with ^87^Sr/^86^Sr ratios above the cut-off value of 0.70909 were trapped typically reached over China, the Korean Peninsula, or western Japan (Figures 3, 4, and S2).

Specifically, terminal points of backward trajectories with short flight duration that started at MS over 26–28 May 2020 were plotted over first-generation outbreak regions such as those in Jiangsu and Shandong provinces, and the Korean Peninsula outside the outbreak region (Figure 3). Typical backward trajectories starting at MS on 28 May 2020 can be seen in Figure S2A. The terminal points correspond to a marked immigration catch peak on 29 May 2020 (29 insects per day; Table S2; Figure 1C). This result suggests that *M. separata* migrated with wind assistance from these regions to Japan. On all dates except 25 May, and 2, 9, 10 June, when *M. separata* adults with high ^87^Sr/^86^Sr ratios (> the cut-off ^87^Sr/^86^Sr) were trapped at MS (Figure 1C), the terminal points of short flight duration were plotted similarly over mainland China, such as in the Jiangsu, Shandong, and Henan provinces (Figure 3). Terminal points for *M. separata* trapped on 25 May and 2 and 9 June were distributed over the East Asian continent as well as western Japan (Figure 3), suggesting a possible migration from those regions. Terminal points for *M. separata* trapped on 10 June were distributed only in western Japan. In addition, on the dates when *M. separata* adults with low ^87^Sr/^86^Sr ratios (below the cut-off ^87^Sr/^86^Sr) were trapped at MS, the terminal points of short flight duration were plotted over mainland China, the Korean Peninsula, and western Japan (Figure S3). These results are discussed in the next section.

Terminal points for the catch on 26 May 2020 at YH with a high ^87^Sr/^86^Sr ratio above the cut-off ^87^Sr/^86^Sr were distributed over eastern China (Shandong and Jiangsu), northeastern China (Liaoning, Jilin), the Korean Peninsula and western Japan (Figures 4 and 1D). For example, a backward trajectory starting at YH on 24 May 2020 reached over the Shandong Peninsula in Shandong province (Figure S2A).

Terminal points for the insects caught at NS in 2020 and 2021 are distributed over the East Asian continent including eastern China, northeastern China, the Korean Peninsula, eastern Russia, and western Japan (Figure 4).

Some terminal points for the catch on 28 May 2020 at YH with a low Sr isotope ratio of 0.70685 (below the cut-off ^87^Sr/^86^Sr) were distributed over southern Kyushu, western Japan (the second from the left on top row in Figures 4A and 1D), suggesting the possibility of a migration from southern Kyushu, where the ^87^Sr/^86^Sr ratio of the reference was low (0.70717 at MN). These trajectory results explain the low Sr isotope ratio of the trapped insect.

## Discussion

The overseas migration pathway of various insect species from China to Japan is one of the major migration pathways in East Asia.^7^^,^^10^^,^^12^^,^^13^^,^^14^^,^^34^^,^^35^ Analyses of the migration for purposes of estimating the migration sources and pathways have so far been based only on trajectory analyses with trapping data. By this method, backward trajectories starting from a trapping site on possible arrival dates are calculated, and their terminal points and valid trajectories suggest the migration source and pathway, respectively. The migration analysis has been successfully applied to many insect species.^14^^,^^34^^,^^35^ However, the evidence of meteorological analysis alone has been circumstantial. In this study we combined the trajectory analysis with an insect’s Sr isotope ratio ^87^Sr/^86^Sr for each trapping event, with the expectation that the Sr isotope ratio of trapped insects could serve as a tracer of their natal origin.

### Validity of the strontium isotope ratio for tracing east–west migration

The basic overwintering and migration ecology of *M. separata* in East Asia can be summarized as follows. The first generation of *M. separata* in China emigrate from the first-generation outbreak region in latitudes between 32 and 36 °N, mainly to northern China, northeastern China, and the Korean Peninsula, in late May to early June.^7^ Since the moth can perform successive multiple night-time flights, the migration destination regions can later become emigration regions.^36^ In Japan, *M. separata* can overwinter in warmer coastal areas of the mainland south of the Kanto district, where the average temperature in January is more than 4°C.^6^^,^^8^ In fact, early trap catches in April and May have been confirmed in the Kansai and Chugoku districts (the western mainland region; roughly <136°E),^8^^,^^37^ and catches have been reported even in March in the southern Kyushu district,^25^^,^^38^ although these catch numbers are very small. It is still unknown whether *M. separata* adults in these districts migrate to northern Japan.

The oriental armyworm cannot overwinter, due to low temperatures in winter in Korea.^10^ A nationwide light trap survey starting on 15 April conducted in Korea from 1987 to 1991 indicated that initial trap-catch events started mid-to late May.^10^ Progeny of the initial insects in May cannot occur in late May to early June due to insufficient accumulated temperature or short time. These catches occurred almost synchronously across the country,^10^ suggesting immigration from outside of the country. The survey suggested that Korea is an arrival area in the migration of the first generation of oriental armyworm from eastern China. Nevertheless, since the oriental armyworm may emigrate for a few consecutive nights (multi-step nighttime migration),^36^ it is apparent that the Korean Peninsula can become a source of a possible subsequent migration step, i.e., a stepping stone.

This study confirmed that the levels of the Sr isotope ratio of the Chinese *M. separata* reference samples and the reference samples from the Japanese immigration areas are largely different. The ratios for the reared insects were correlated with those for the host plants in the two countries, or different geological ages of the Chinese mainland’s soil and Japanese soil in the immigration areas as a basis (Figure 1B and the Sr isoscape by Bataille et al; ^24^). For the overseas immigration events in Japan, therefore, the Sr isotope ratio of 0.70909, the cut-off value of the logistic regression curve, was selected as a *tentative* boundary level for the local population in the immigration areas. Thus, this *tentative* threshold should not be considered representative for the whole Japanese population. When the ratio of an initial immigrant insect in the early summer is found to be above the threshold, the insect’s natal origin can be assumed to be China or somewhere outside the immigration area. When the ratio is less than or equal to the threshold, the insect can be assumed to be of local origin or from someplace with a low ^87^Sr/^86^Sr ratio. Note that there is some uncertainty in the precise cut-off level that should be used to predict the natal origin, and that there are areas with high Sr isotope ratios in other parts of Japan.^24^ The local Japanese populations occurring in such high ratio areas are carefully discussed below.

In the main immigration season of the first-generation, 46 adult individuals caught at MS were found to have Sr isotope ratios higher than those of the Japanese reference insects in the immigration areas, and those high ratios fell within the Chinese reference ratio range (Figure 1), suggesting that those insects might have originated outside of the immigration area.

In addition, the marked catch peak appeared at MS on 29 May 2020 when westerly winds blew at the site (Table S2). The terminal points of the associated backward trajectories distributed over the first-generation outbreak region and the Korean Peninsula (Figure 3). All these pieces of evidence, including the trapping, flight trajectory and Sr isotope ratios of the immigrants, therefore suggest that overseas migration from eastern China or the Korean Peninsula occurred. Interestingly, the former pathway is the same as that of the small brown planthopper, *Laodelphax striatellus*.^39^ Regarding the latter pathway, because the peninsula is one of the destination areas of the *M. separata*’s seasonal migration,^7^^,^^10^ our results suggest a multi-step migration from eastern China via the peninsula to western Japan. This is the analysis in which the Sr isotope ratio of the insect body was successfully applied to analysis of the overseas east–west migration arriving in Japan. The Sr isotope information enhanced the accuracy of migration analysis by providing the individual natal information of the insects.

The isotopic analysis consequently indicated that the trap catches at MS might be a mixture of local individuals and overseas immigrants. It highlights the value of using the isotopic tracer in migration study. The result could not be achieved without the isotopic information of the insects, demonstrating the analytical advantage conferred by this data.

In all the other trapping cases at MS except those on 25 May and 9 and 10 June 2020, backward trajectories from the monitoring sites reached over the first-generation outbreak region of China, the Korean Peninsula, northeastern China, and eastern Russia (Figure 3), suggesting migrations from the East Asian continent like the above.

However, in the trapping cases at MS on 25 May and 9 and 10 June 2020, some backward trajectories reached over western Japan. According to the bioavailable Sr isoscape of Bataille et al.,^24^ about 90% of the location points in the Chugoku and Kansai districts of western Japan have ^87^Sr/^86^Sr ratios greater than the ^87^Sr/^86^Sr cut-off value. In addition, trap catches were reported in April or May in those districts, as described above, although the numbers were very small. Therefore, it is not feasible to determine the natal origin and either the overseas or domestic migration pathway based on the current method for those catches at MS. Hence, the isotopic and trajectory analyses for the trap catches at MS are mostly consistent with each other with a few undeterminable cases.

Likewise, 10 adult individuals caught in northern Japan were found to have Sr isotope ratios higher than the cut-off ratio, and those high ratios fell into the Chinese reference ratio range (Figure 1D). However, since the terminal points of the backward trajectories are distributed over the East Asian continent as well as the Japanese Chugoku and Kansai districts, where high Sr isotope ratios are expected, two possibilities are suggested regarding the origin of the trapped insects: the immigrants arrived from the first-generation outbreak region, including the migration waypoint regions on the East Asian continent, or they arrived from western Japan, where the Japanese first generation may have occurred with high Sr isotope ratios. Our present analysis does not allow us to state which of these origin scenarios is more likely.

For an individual insect caught on 28 May, 2020, at YH, its isotope ratio (0.70894) is lower than that for Akita prefecture (Figure 1D), the possible source may be the southern Kyushu district (Kagoshima prefecture) (see the terminal point map for 28 May in Figure 4A).

Therefore, our migration analysis method combining the Sr isotope ratios of insects with a conventional trajectory analysis successfully, if only to a limited degree, revealed the overseas migrations of insects from the East Asian continent to Japan on an individual basis, providing one piece of a vivid migration image.

Our present study focused on the geological age of soils on the East Asian continent and the Japanese immigration areas and elucidated the eastward migration across the East China Sea and the Sea of Japan. The results indicated that at least portions of the eastward migrations of *M. separata* traveling at a limited latitudinal range were more successfully traced with the ^87^Sr/^86^Sr ratio than by previously available methods. Hence, the method was found to be successfully applicable to immigrants in the Kyushu district but could not determine the origin and pathway for the immigrants caught in northern Japan due to a lack of information to discriminate Chinese overseas and Japanese domestic immigrants. Alternative approaches may be needed to further improve the analytical quality of migration studies, such as using multiple elements in isotope analysis or using element analysis like a combination of the isotope ratio and Sr concentration.^26^^,^^30^

In Henan province, the ^87^Sr/^86^Sr ratios for winter wheat ‘grain’ collected at Xinxiang (XX) and Huixian (HX) (Figure 1A) were previously reported to range from 0.71080 to 0.71150 (3 varieties × 2 years × *n* = 18), and from 0.71099 to 0.71148 (3 varieties × *n* = 9), respectively.^40^^,^^41^ The ^87^Sr/^86^Sr ratio of the wheat leaves collected in the province (0.71146–0.71294) seemed to be slightly higher than that of the wheat grains. However, the Sr ratio of wheat leaves at XX was 0.71146, which was within the range of the ratio for the XX grain. The other locations used for the collection of wheat leaf samples were in the southern part of the province, whereas HX and XX, the sites used for the collection of the grain samples, were in the northern part. Therefore, these differences in location might have contributed to the disparate Sr isotope ratios of wheat in the province. Alternatively, this could be due to differences in agricultural practices and sources/types of products used to manage the land between the north and south parts. This study also found that the ^87^Sr/^86^Sr ratio of the wheat leaves differed between the Henan and Jiangsu provinces, supporting the ideas put forward above.

On the other hand, the accurate boundaries or degree of overlap among the Chinese source areas and Japanese immigration areas corresponding to the Sr isotope ratios of insects are not yet known, because the sample size and the number of locations of the Chinese reference data are limited compared to those of the wide source region in China; there were no insect reference data in Jiangsu province and there were no samples from Shandong province, which is another major possible source.^7^ It was also uncertain why there was a large variance in the Henan’s insect reference data.

### The ^87^Sr/^86^Sr ratio of agricultural soils

We investigated the Sr isotopes of the samples only from the agricultural fields. Since *M. separata* is an insect pest whose hosts are mostly crop plants and pastures, on which larvae are easily found, our results should be representative of the insect’s Sr isotope situation in nature. However, it should be noted that the ^87^Sr/^86^Sr ratio of agricultural soils, or of host plants grown in the agricultural soils, is generally affected by agricultural materials such as fertilizers, chemicals, additional soils outside, and animal manure (or originally animal foods),^24^^,^^26^^,^^42^ and is basically not predictable, although the bioavailable ^87^Sr/^86^Sr ratio of *natural* soils has been predicted globally.^24^ Therefore, this study didn’t perform likelihood assignment of natal origin on the Sr isoscape. Instead, migration source was estimated with trajectory analysis.

Regarding the unpredictability of the ^87^Sr/^86^Sr ratio of agricultural soils, the relationship between the ^87^Sr/^86^Sr ratios of the reference host and the bioavailable Sr isoscape^24^ is shown in Figure S8. The data used are listed in Table S9. Although the plots roughly follow the linearity line (in red), the slop of the regression line in blue is small (0.32), and R^2^ value is 0.079. Large variation was observed around the regression line, indicating partly the effect of agricultural materials.

On the other hand, local areas often use standard agricultural practices. Farmers’ agricultural materials applied to their fields such as fertilizers, chemicals, and animal manure (originally from animal feeds) are generally supplied by a limited number of suppliers. This situation suggests that the ^87^Sr/^86^Sr ratios of plants and insects on such fields could indicate a certain representative distribution for the area, even if the ^87^Sr/^86^Sr ratio of an individual field is unpredictable.

As an example, Figure S9 shows the ^87^Sr/^86^Sr ratio of leaves of feed maize collected from fields extending in a 10-km range in Koshi city, Kumamoto prefecture, Japan, where dairy farms comprise major agricultural farming (Table S8). The geological map of the area shows that bedrocks of the sampling sites are sedimentary (green) or igneous (pink) rocks in Cenozoic Quaternary under the influence of a volcano, Mt. Aso (Figure S10)), which predicts low ^87^Sr/^86^Sr ratios. Figure S9 shows that the ^87^Sr/^86^Sr ratios distribute less than 0.70900 with a large variation (0.70600–0.70900). This variation may be caused by this area’s agricultural materials (agricultural practices).

Furthermore, the ^87^Sr/^86^Sr ratio of the Solanaceae leaves from the farmers’ fields distributes similarly less than 0.70935 (Figure S7).

It is important that these ^87^Sr/^86^Sr ratio ranges correspond to that of the reference values in Kyushu and Tohoku districts (Figure 1B) whose bedrocks are Cenozoic rocks.

Based on these points, it can be said that the expected ^87^Sr/^86^Sr ratio range of agricultural soils in the study area is mostly predictable. The ^87^Sr/^86^Sr ratios of hosts in agricultural fields could thus be used in our study as references for agricultural insect pests.

It is also necessary to note that the Sr isotope ratio of the two insects in LY and XY exhibited very low values, 0.70723 and 0.70415, respectively (Figure 1B). Regarding such low Sr isotope ratios in an agricultural field, it has been recently recognized that the application of fertilizers and agricultural limes (calcium carbonate) and influences the ^87^Sr/^86^Sr ratio of the soil-exchangeable fraction.^24^^,^^26^^,^^42^ The lowest value of 0.70415 may be too low (−0.00673 from the Chinese insect’s average) to explain with lime, as a decrease of the ^87^Sr/^86^Sr ratio of surface soil applied with lime has been reported about 0.0037.^26^ Presumably, unevenly applied fertilizers in the agricultural fields in Henan province might have affected the soil and wheat, and thereby the ^87^Sr/^86^Sr ratios of the insects collected there. The reason for the very high ratio at LH is also unknown. These are future subjects to be considered.

### Historical aspect of the study and future directions

The migration of rice planthoppers (the brown planthopper, *Nilaparvata lugens* Stål, and the white-backed planthopper, *Sogatella furcifera* Horváth), major migratory insects, over the East China Sea has been intensively investigated following the observation of planthoppers on a weather observation ship sailing far from land in the Pacific Ocean,^43^ which suggested that even such tiny insects can fly long distances overseas. Marking experiments for *S. furcifera* were conducted by spraying dye on Chinese local emigrants in 1985 and 1986 in a cooperative scientific project between China and Japan.^44^ Unfortunately, the survey resulted in unsuccessful recapture of any marked *S. furcifera* individual in Japan.^44^ The present study, thus, successfully presents direct isotopic evidence of the Chinese natal origin of immigrant *M. separata* caught in Japan.

There are many other migratory insects of Chinese natal origin crossing the East China Sea and the Sea of Japan, such as rice planthoppers,^43^^,^^44^ *Spodoptera litura* Fabricius,^35^ *Spodoptera frugiperda* J. E. Smith,^34^ the oriental fruit fly *Bactrocera dorsalis* Hendel,^45^ and the rice leaf roller *Cnaphalocrocis medinalis* Guenée.^46^ Our method should be applicable to them as well. The combination of the Sr isotope ratio and trajectory analysis can also contribute to elucidation of the migration traveling between areas of different Sr-isotope signatures. Insect migration between Australia and New Zealand would be another such example.^29^^,^^47^

### Limitations of the study

The selected Japanese reference sites were located in major immigration areas in the Kyushu district, in the Tottori prefecture of the Chugoku district, and in the two prefectures of the Tohoku district, where it so happens that all the reference Sr isotope ratios fall into the lower range. However, there are areas showing high Sr isotope ratios in Japan that overlap with the Chinese isotope ratio range. When backward trajectories estimate only one source area—i.e., the East Asian continent or the Japanese area of high isotope ratios—the migration source can be determined. This is the successful MS case. However, when the trajectories reach over both the areas, the uncertainty in the estimation cannot be resolved. The latter case needs additional information. Another future direction of study might be to develop an additional multimodal analysis, such as a combination of the current method and other isotopic or elemental information collected from the migrant’s body.

## Resource availability

### Lead contact

Further information and requests for resources and reagents should be directed to and will be fulfilled by the lead contact, A.O. (aotuka@affrc.go.jp).

### Materials availability

This study did not generate new unique reagents.

### Data and code availability

• Data: Strontium isotope ratio data are available in Table S9 in the supplemental information.
• Code: The shell script codes and the C codes for trajectory analysis are available in Programs 1–3 in the supplemental information.
• Any additional information required to reanalyze the data reported in this study is available from the lead contact upon request.

## Acknowledgments

This study was supported by Strategic International Collaborative Research project promoted by the Ministry of Agriculture, Forestry and Fisheries, Tokyo, Japan [JPJ008837]; the Joint Research Grant for the Environmental Isotope Study of Research Institute for Humanity and Nature, Japan; the National Natural Science Foundation of China, China [32072414]; and Science and Technology Innovation Team project of Henan Academy of Agricultural Sciences, China [2021TD14, 2022TD13, 2023TD13, 2024TD30]. Authors thank Naoki Kato for providing the reference insects and pasture collected at Kimotsuki, Ken Oikawa of Aomori prefecture for supplying with the local larvae and their food leaves, Yuuki Ikenoue of Kagoshima Prefecture for supplying maize leaves, Sayuri Gyotoku for rearing the insects for the experiment, and Hiroyuki Itoh for managing maize fields. Authors thank Chinese and Japanese farmers who gave permission to collect samples in their field.

## Author contributions

A.O. conceived the study. N.H., G.A., A.O., and T.N. designed and conducted the breeding experiments in Japan. A.O. directed the trap monitoring in Nagasaki prefecture. G.A. collected and established the successive strains in Japan. H.F. and G.L. designed and directed experiments in China. S.Z. collected field insect samples and S.L. conducted the breeding experiments in Luohe, Henan province. C.T. collected host plant samples and directed isotope analysis in China. T.N. directed the monitoring in Akita prefecture. G.H. directed the wheat-leaf collection in Jiangsu province. N.H., A.O., I.T., and K.-C.S. designed and conducted the Sr isotope analysis in RIHN. A.O. performed the trajectory analysis. C.T., A.O., and H.F. wrote the manuscript. N.H., C.T., and A.O. revised the manuscript. All authors edited and approved the final manuscript.

## Declaration of interests

Author G.H. is guest editor of the special issue “Insect Migrations” in which the manuscript will be included. The authors declare no competing interests.

## STAR★Methods

### Key resources table

**Table:** 

REAGENT or RESOURCE	SOURCE	IDENTIFIER
**Biological samples**		

*Mythimna separata* male adults trapped in Nagasaki prefecture	National Agriculture and Food Research Organization (NARO)	*M. separata* at MS (Figure 1A; Table S1)
*Mythimna separata* male adults trapped in Akita prefecture	Akita Plant Protection Station	*M. separata* at NS and YH
*Mythimna separata* larvae collected in Aomori prefecture	Aomori Plant Protection Station	*M. separata* at SH and GG
*Mythimna separata* larvae collected in Akita prefecture	Akita Plant Protection Station	*M. separata* at NS
*Mythimna separata* larvae collected in Kagoshima prefecture	NARO	*M. separata* at KT
*Mythimna separata* larvae collected in Henan province	Luohe Academy of Agricultural Sciences	*M. separata* at LH
*Mythimna separata* pupae collected in Henan province	Henan Academy of Agricultural Sciences	*M. separata* at LY and XY
*Zea mays* L. leaves as diet collected in Japan	NARO	Maize at KU, GG, UZ, KS, TH, MJ, and MN
*Triticum aestivum* L. as diet collected in Aomori prefecture	Aomori Plant Protection Station	Wheat at SH, GG
*Dactylis glomerata* L. as diet collected in Akita prefecture	Akita Plant Protection Station	Orchard grass at NS
*Dactylis glomerata* L. as diet collected in Kagoshima prefecture	NARO	Orchard grass at KT
*Triticum aestivum* L. leaves as host plant collected in Jiangsu province	Nanjing Agricultural University	Wheat at LYG, YC, TZ and NT

**Chemicals, peptides, and recombinant proteins**		

Sr standard, NIST SRM987	National Institute of Standards and Technology, USA	https://shop.nist.gov/ccrz__ProductDetails?sku=987
MCI GEL CHP20P	Sigma-Aldrich, Merck KGaK, Germany	https://www.sigmaaldrich.com/
Eichrom Sr-Spec resin	Eichrom Industries, USA	https://www.eichrom.com/eichrom/products/
Sr Spec resin SR-B25-S	TRISKEM International	https://www.triskem-international.com/

**Experimental models: Cell lines**		

Japanese *Mythimna separata* laboratory strain	The Koshi Research Station, NARO	Strain collected from maize plots at the Koshi Research Station, NARO (32.88°N, 130.74°E) in June 2020

**Software and algorithms**		

Home-made shell script and C programs for trajectory analysis	This manuscript	See Programs 1–3 in the supplemental information

**Other**		

Thermal ionization mass spectrometer Triton	Thermo Fisher Scientific K.K., Tokyo	https://www.thermofisher.com/
Thermal ionization mass spectrometer Triton XT	Thermo Fisher Scientific K.K., Tokyo	https://www.thermofisher.com/
Thermal ionization mass spectrometer Isoprobe-T	GV Instruments (now Isotopx Ltd, UK)	https://www.isotopx.com/about-us
Class-1000 clean room	Research Institute for Humanity and Nature, Japan	N/A
Class-1000 clean laboratory	National Institute of Metrology, China	N/A

### Experimental model and study participant details

#### Collection of reference host plants and insect populations

To obtain a ref. ^87^Sr/^86^Sr ratio for *M. separata* adults occurring locally in China and Japan, the Sr isotope ratios of host plants grown in the two countries and moth adults emerged by feeding on the local host plants were measured. In Japan, as a diet resource for larvae, fresh leaves of gramineous pasture, wheat, and maize were collected from May to July 2021 at 10 locations in 8 prefectures (Table S1; Figure 1A).

In China, *M. separata* pupae were collected in two wheat fields in Henan province in mid-May 2021 (Table S1; Figure 1A). A single wheat plant at the flowering stage was also collected in 10 locations in 2 provinces in May 2022 (Table S1).

#### Japanese host plants

Eight Japanese prefectures where diet sources were obtained are Aomori, Akita, Tottori, Fukuoka, Nagasaki, Kumamoto, Miyazaki, and Kagoshima (Table S1; Figure 1A). Aomori and Akita prefectures are in northern Japan, where *M. separata* immigrants arrive in early summer. The other 6 prefectures are in western Japan, where daily and livestock farming is active and maize for feed is cultivated. Tottori and Nagasaki prefectures are also known as an immigration area in early summer. Three to five young maize (*Zea mays* L., cv. not identified) plants of about 1-m height, and young pasture (*Dactylis glomerata* L., cv. not identified) and wheat (*Triticum aestivum* L., cv. not identified) leaves in quantities sufficient for feeding were collected from a farmland at each site. The samples in a clean plastic bag were sent to Koshi Research Station (KS), National Agriculture and Food Research Organization (32.88°N, 130.74°E) and kept fresh in a large refrigerator.

The surface of a maize leaf for reference was cleaned by wiping with wipe papers soaked with 70% ethanol for a few times not to cause diseases of rearing insects. Maize leaves cleaned in the same way were used for isotope analysis as well. Wheat leaves (SH, GG, LYG, YC, TZ and NT) and orchard grass (NS and KT) for rearing and isotope analysis were not cleaned.

#### Japanese breeding experiment

Successive strains of *M. separata* were established from more than 100 5th- or 6th-instar larvae collected from maize plots at the Koshi Research Station (KS) in June 2020. Larvae hatched from an egg mass were reared until the 3rd-instar stage in a clear plastic cup of 129 mm in diameter × 44 mm h (Biocup, FG320TCL; Risu Pack Co.) for each site. From the 4th-to 6th-instar stage, 30 well-grown larvae were selected and each of them was individually reared in a clear plastic cup of 66 mm in diameter × 28 mm h (clean cup, 60TCL; Risu Pack Co.). After pupation, residues of the food leaves were removed. The adults were maintained without any food until the third day after emergence, and then kept in a freezer at −20°C for isotope analysis. All the indoor insect strains were reared in a growth chamber (PU-30, 2087w×3316 d × 2500 h mm; Koito Manufacturing Co.) at 25°C under a 16-h light/8-h dark condition.

Fourth-to 6th-instar larvae were collected at random in a pasture (*Dactylis glomerata* L., cv. not identified) field at Kimotsuki (KT) in Kagoshima prefecture in June 2020 and were reared to adults in a laboratory of the Koshi Research Station with pasture leaves from the same field (Table S1).

Fourth-to 6th-instar larvae were collected in wheat fields at Shichinohe (SH) and Goshogawara (GG) in Aomori prefecture in June 2021 were reared in a laboratory of the Koshi Research Station along with wheat leaves from the corresponding site until emergence (Table S1). These larvae were placed individually in a clear plastic cup of 66 mm in diameter × 28 mm h until pupation. Rearing temperature and light conditions were the same as described above.

Fourth-instar larvae were collected in a pasture (*Dactylis glomerata* L., cv. not identified) field at Noshiro (NS), Akita prefecture in June 2021, and the larvae were reared with orchard grasses obtained from the same field until adult emergence under a natural condition in a laboratory of the Akita Plant Protection Station. Only one adult sample was successfully obtained at NS. The adult insect samples were subjected to isotope analysis.

#### Immigration monitoring and sampling for isotope analysis

Molasses traps (or food traps) of a dry type were used at Yurihonjo (YH) and Noshiro (NS) in Akita prefecture, northern Japan. The molasses in a tray inside the trap was covered with a mesh, so that a moth was trapped without eating the molasses. A search light trap was used at Minami-Shimabara (MS) in Nagasaki prefecture, western Japan, to monitor immigrant *M. separata* adults (Tables S2 and S3; Figure 1A). The northern and western prefectures were selected to analyze different immigration pathways. The trap data from 20 May to 20 June in 2020 and 2021 were selected in Akita prefecture, targeting the main immigration season of *M. separata*’s first generation. Two years were used for the investigation in northern Japan due to the relatively small sample number in this region. The trapping events in 2020 in Nagasaki prefecture was selected because a clear catch peak was evident in a preliminary evaluation (Table S2). Other monitoring parameters are described in Table S3. The insects were morphologically identified by the authors and sometimes with the aid of a stereomicroscope (S8APO, Leica Microsystems).

The method used to collect samples from trapped insects for isotope analysis differed between Akita and Nagasaki prefectures. We considered that trapped adults might have been immigrants that had arrived in Akita prefecture, because the prefecture is a non-overwintering area. All samples per collection date were selected in Akita prefecture. On the other hand, adults trapped in Nagasaki prefecture in early summer were expected to be a mixture of local Japanese individuals and overseas immigrants. All adults caught on 29 May 2020, when a large catch peak appeared, and on other collection dates on which there were <10 catches were used for isotope measurement. Otherwise, 5 individuals were randomly selected from among the adults trapped on dates with ≥10 catches.

#### Chinese breeding experiment

In China, *M. separata* pupae were collected in wheat (*Triticum aestivum* L., cv. not identified) fields in Luoyang (LY) and Xinyang (XY), Henan province in mid-May 2021 (Table S1; Figure 1A). The pupae were found above or below surface soils (<5 cm depth).

Larvae were reared on wheat (*Triticum aestivum* L., cv. not identified) seedlings cultured in a laboratory until early 3rd instar and subsequently on winter wheat (*Triticum aestivum* L., cv. not identified) in the field to pupa in Luohe (LH), Henan province in May 2021 (Table S1; Figure 1A). The province is a part of the first-generation outbreak region. All the pupae were kept in a plastic box without the soils at 26.5°C until emergence in a laboratory of the Henan Academy of Agricultural Sciences. Adults were maintained without food until the third day after emergence and kept in a freezer at −20°C until isotope analysis.

### Method details

#### Strontium isotope analysis

The ^87^Sr/^86^Sr ratios of the samples reared and collected in Japan and China were analyzed in Japanese and Chinese institutions (The Research Institute for Humanity and Nature (RIHN), Kyoto, Japan, and the Institute of Chemometrics and Analytical Science (ICAS), the Chinese Academy of Metrology, Beijing, China), respectively. A basic analytical process including acid digestion of the samples, column Sr separation, and measurement of the ^87^Sr/^86^Sr ratios with a thermal ionization mass spectrometer is similar between the laboratories with minor methodological differences. To render the data from the two countries comparable, the same correction method and standard material were used. Blank tests are found below.

The bodies of *M. separata* adults, and the leaves of pasture, wheat and maize were dried at 60°C for 12 h in a dry heat sterilizer (KM-300V from TAIYO Co., Japan or FXB-3 from Shanghai Kuncheng Scientific Instrument Co., China).

Methods of sample preparation and isotope measurement differ slightly among different countries. In Japan, all body parts except the abdomen of the trapped insects were subjected to acid digestion. The abdomen was removed to minimize the possibility that food taken by the immigrants after their arrival in Japan would influence the isotope analysis. Whereas body parts except the abdomen of the reared reference insects were used in the initial analysis, the remaining abdomens or new whole bodies were used for later analyses. The parts used were changed in the middle of a measurement series when some of the initial samples were lost by accidental contamination at sample loading on tungsten filament. Since the reference insects had no food after emergence, the Sr isotope ratios of the different parts were expected to be similar at each site. Whole bodies were used to increase the Sr signal intensity.

Each dried insect sample (avg. 25 mg) was placed in a 7-mL TFM insert vial (product number: 40133; Milestone Co., USA) with a mixed solution of 0.9 mL of 15M HNO_3_ (Ultrapure-100; Kanto Chemical Co., Tokyo) and 0.1 mL of 35% H_2_O_2_ (TEMAPURE AA-10; Tama Chemicals Co., Tokyo), and then digested using a microwave digestion instrument in an ordinal laboratory room (ETHOS ONE Microwave, Milestone Co., Tokyo). The digestion recipe used was two cycles of 10-min of heating at 180°C. After evaporation at 180°C, the digested residue was dissolved again with 1.0 mL of 3.5 M HNO_3_ (double distilled electronic grade) and 0.1 mL of 35% H_2_O_2_ and the solution was transferred into a 7-mL Teflon (perfluoro alkoxyl alkane) vial with a lid, then placed on a hotplate to heat at 140°C for more than 7 h. The resulting solution was again evaporated to dryness at 140°C.

Leaves of the host plant were cut into small pieces <2 × 2 mm with ceramic scissors. Cut leaves of 200 mg were similarly digested with the microwave digestion instrument heating at 200°C for 20 min. The supernatant solution was transferred into a Teflon (PFA) vial and evaporated to dryness at 180°C. The digestion protocol was identical to that used for digestion of the insect bodies. All digested samples were dissolved with 0.3 mL of 3.5M HNO_3_ before Sr chemical separation.

A hand-made PFA column (i.d. = 3 mm, resin height = 16 mm) with MCI GEL CHP20P (Sigma-Aldrich; Merck KGaK, Darmstadt, Germany) and Eichrom Sr-Spec (Eichrom Industries, Darien, IL) were used. After resin setting in a clean room, the column was washed sequentially with 2.0 mL of 7M HNO_3_, 0.5 mL of Mill-Q ultrapure water, 2.0 mL of 6M HCL and 0.5 mL of Mill-Q water. To adjust the ionic condition of the resins, 2 mL of 3.5M HNO_3_ was applied, then 0.3 mL of the sample solution was loaded into the column. Major cations and Ba in tested samples were washed out from the column with 0.5 mL of 3.5M HNO_3_ and subsequent 0.5 mL of 7M HNO_3_. Sr fractions were collected with 1.8 mL of 0.05M HNO_3_ receiving in a 3-mL Teflon vial. The resin was used only once. The Sr extraction solution was evaporated at 90°C overnight.

All procedures involved in acid addition to the samples, evaporation, acid digestion on the hotplate, and column separation were carried out in a class-1000 clean room at the Research Institute for Humanity and Nature (RIHN), Kyoto. The first acid digestion with the microwave instrument was conducted in a regular laboratory room.

The Sr stable isotope ratio ^87^Sr/^86^Sr was measured on a thermal ionization mass spectrometer (TIMS), or Triton and Triton XT thermal ionization mass spectrometer (Thermo Fisher Scientific K.K., Tokyo) at RIHN. A sample containing Sr of 10–20 ng was loaded onto a degassed single tungsten filament (H. Cross Company, Moonachie, NJ) with a tantalum-oxide activator (sample loading). Five Sr isotopes, ^84^Sr to ^88^Sr, were measured in the static multicollection mode using five faraday cups connected to each 10^11^-Ω amplifier (Triton). Fourteen insect samples from Akita prefecture, 31 insect samples trapped on 29 May and 12 June 2020 in Nagasaki prefecture, and 44 reared insects and their host leaves were measured. A single measurement consisted of 100 cycles (10 cycles/block × 10 blocks) of 8.4-s signal integration. The mass fractionation was corrected using ^86^Sr/^88^Sr = 0.1194 by the exponential law. All measured sample ratios were normalized to the ^87^Sr/^86^Sr value of a standard NIST SRM987 (concentration: 145 ppm; volume: 2.0 μL onto a W filament), i.e., 0.71025.^48^ The mean ratio of the standard measured by Triton was 0.710231 ± 0.000011 (*n* = 42).

Similarly, 70 insects trapped in Minami-Shimabara on any day between 6 May and 24 June 2020 except the two days above (29 May and 12 June) were measured with a Triton XT whose five faraday cups were connected to two 10^11^-Ω amplifiers for ^84^Sr and ^88^Sr and three 10^13^-Ω amplifiers for ^85^Sr to ^87^Sr. A single measurement consisted of 150 cycles (15 cycles/block × 10 blocks) of 8.4-s signal integration. The mean ^87^Sr/^86^Sr value of the NIST SRM987 standard was 0.710242 ± 0.000013 (*n* = 19) by Triton XT, and the measured ratio of the sample was adjusted to the recommended value for the NIST SRM987 standard (i.e., 0.71025).

A blank test in the RIHN’s clean room was performed as follows: totally 5 blanks were inserted at a rate of one blank among 9 or 10 insect samples. Forty-six samples in total were tested. The blanks in the cleanroom operation from acid digestion, evaporation to dryness, second digestion, second evaporation, and to column separation were treated the same way as the samples. For the blanks, 0.4 mL of 3.5N HNO_3_ were added to each blank vial and 0.1 mL of the solution was taken out for element analysis. The rest of the blank’s solution was applied to column separation. For the samples, 5.0 mL of 3.5N HNO_3_ were added to each sample vial and 0.1 mL of the solution was taken out for element analysis. Strontium concentration of the blanks and samples just before the column separation were measured with an ICP-MS (Inductively Coupled Plasma Mass Spectrometry) instrument. The result showed that an average of Sr concentration of the samples (insects without forewings and abdomen) and the blanks were 7.278 ng/insect and 0.175 ng/blank (Table S4), respectively. After column separation of the blanks, half of 2mL solution was applied to the second ICP-MS measurement and the rest were dried out for the isotope ratio measurement. Strontium in all the five blank vials (totally 0.272ng) (Table S5) was loaded on one filament and the ^87^Sr/^86^Sr ratio were measured to get an average ratio value. The result indicated the average ^87^Sr/^86^Sr ratio of the blanks was 0.70905 (standard error 0.000190).

Based on the average concentration of the blanks and samples and the average ^87^Sr/^86^Sr ratio of the blanks, a contamination error can be estimated. For example, when a true ^87^Sr/^86^Sr ratio of a sample is 0.71000, measured ^87^Sr/^86^Sr ratio and error in our measurement environment will be 0.70998 (=(0.71000 ∗ 7.278 + 0.70905 ∗ 0.175)/(7.278 + 0.175)) and −0.00002, respectively. Similarly, the general contamination error for the Japanese measurement was estimated (Figure S4). The result indicated that the level of contamination is low enough for the ^87^Sr/^86^Sr ratio measurement of oriental armyworm (Sr of 7.278 ng in average).

In China, sample preparations and isotope measurements of *M. separata* and wheat samples collected in Henan and Jiangsu provinces, China were conducted at ICAS. For each adult *M. separata* sample, all body parts except the abdomen were placed in a PFA vial tube, and then 3 mL double-distilled HNO_3_ and 0.5 mL 30% H_2_O_2_ were added in sequence. The PFA vials were heated on a hot plate at 200°C for 12 h and evaporated to dryness. Wheat leaves, which were not cleaned, were dried at 65°C until reaching a constant weight, and then about 200 mg per sample was digested in the same way. Sample purification was carried out in a Class-1000 clean laboratory (National Institute of Metrology, China). Acid-cleaned glass columns (4–6 mm in diameter) were filled with 130 mg Sr Spec resin (SR-B25-S, particle size: 50–100 μm; TRISKEM International) in order to eliminate potential interference elements. The optimized purification process is shown in Table S6. The elute solution was evaporated to dryness and dissolved with 30 μL 2% HNO_3_ before loading on a single degassed Re filament. The ^87^Sr/^86^Sr ratio of the samples was measured with thermal ionization mass spectrometry (TIMS) (Isoprobe-T; GV Instruments) in static multi-collection mode. Five Faraday Cups Axial, H1, H2, H3, H4 and H5, were used to collect isotopes ^84^Sr, ^85^Rb, ^86^Sr, ^87^Sr, and ^88^Sr, respectively. The potential Rb interference was burned during the heating program. In addition, the ^87^Rb signal was subtracted by monitoring the ^85^Rb intensity. The mass fractionation was corrected using ^86^Sr/^88^Sr = 0.1194 by the exponential law. The mean ^87^Sr/^86^Sr value of NIST SRM987 measurements was 0.710262 ± 0.000012 (2σ, *n* = 9). The measured Sr ratio of the sample was finally adjusted to the standard’s recommenced value of 0.71025^48^ by using the mean measured ratio of the standard.

A blank vial was processed from acid digestion, evaporation to dryness, to column Sr separation, in the same way as the Chinese samples in the clean room of ICAS. Strontium concentration in the blank was found 0.37 ng. The contamination level was similarly evaluated, indicating the level was low. (Figure S5).

In the sample preparation, we did not perform nitrogen-gas cleaning to blow particles from the surface of the *M. separata* sample,^29^ even for trapped insects. This was because the samples would later be applied to lead (Pb) isotope analysis, and a preliminary ICP-MS (inductively coupled plasma mass spectrometry) measurement of 3 *M separata* trap samples suggested that at least a small amount of lead particles was present on the body surface of the insects.

In lieu of cleaning the tested trap-catch samples, the effect of the cleaning on the ^87^Sr/^86^Sr ratio was evaluated as follows. Twenty, 9 and 9 insects trapped at MS, NS and Gojome, respectively, were used for the evaluation (Figure S6; Table S9). Each dried sample without abdomen and forewings was cut in two along the central body axis with a 40 kHz ultrasonic cutter (ZO-30PII with an RR02 blade; Echo Tech Inc., Toyohashi, Japan). One-half of the body was placed in a plastic mesh bag and cleaned twice using a nitrogen gas flow of 350 kPa for 30 s each time under laboratory conditions. The other half was not cleaned. The ^87^Sr/^86^Sr ratios of the total 38 pairs at 3 sites were measured with the Japanese isotope measurement protocol described above. The results indicated linearity between the cleaned and uncleaned samples (y = 1.0051x-0.0035, R^2^ = 0.75) (Figure S6). The standard deviations of the difference between the ^87^Sr/^86^Sr ratios of the cleaned and uncleaned halves of the body was relatively small at 0.00039.

Regarding cleaning of the leaves for isotope analysis, since larvae eat the leaves including the surface and attached dusts at the same time, non-cleaned leaves could indicate the ^87^Sr/^86^Sr ratios close to real ones. As reference, the ^87^Sr/^86^Sr ratio of cleaned and uncleaned leaves of Solanaceae plants were previously measured (Figure S7; Table S9), although they are not Poaceae plants. A part of a leaf or leaves were dipped into ultra clean water in a clean beaker and cleaned with ultrasound vibration applied. The result indicated linearity between the two groups (Figure S7). Standard deviation of difference between the two groups was 0.00028. In addition, the ^87^Sr/^86^Sr ratios of the Japanese uncleaned leaves (the wheat and orchard grass leaves) overlap with the ^87^Sr/^86^Sr ratios of the cleaned maize leaves in the measured ratio range. The ^87^Sr/^86^Sr ratios of the Chinese uncleaned wheat leaves overlap with the ^87^Sr/^86^Sr ratios of reared insects. The results suggest the isotope measurement of the reference host plants (Figure 1B) should be reliable.

#### Trajectory analysis

Backward trajectory analysis was conducted to estimate the migration source and pathway from the source to Japan. Backward trajectories from the trap sites were calculated using a Windows-based public version of the HYSPLIT (Hybrid Single-Particle Lagrangian Integrated Trajectory) model developed by the Air Resources Laboratory, National Oceanic and Atmospheric Administration (NOAA), Boulder, CO.^49^ The meteorological grid data used were NOAA’s Global Data Assimilation System Data with a horizontal resolution of 1 × 1° and a temporal resolution of 3 h (archive information available at https://www.ready.noaa.gov/gdas1.php). Five conditions were established with respective differences in flight duration, starting height, flight speed, ambient air temperature, and possible immigration dates and used for the trajectory calculation described below. Backward trajectories were calculated with the program *hyts_std* of the HYSPLIT system (Program 1 in the supplemental information). To estimate the source area, the frequency of valid terminal points of backward trajectories in the analytical period (3–9 days; Table S7) was counted in each grid of 0.25 × 0.25° with the program *trajfreq* and mapped with the program *concplot*. Plots over the seas, which was not a source, were removed (Program 2 and 3 in the supplemental information).

#### Conditions for the trajectory calculation are as follows

##### Flight duration

Noctuid moths in the continent generally begin to take-off around dusk, keep flying during the night, and terminate their flight at the following dawn.^34^ In this study, since *M. separata*’s exact arrival time in the destination was not known, 24 backward trajectories at 1-h intervals for a date of interest were calculated with the starting time of 23.00 h Coordinated Universal Time (UTC), or 08.00 h Japan Standard Time (JST), to 00.00 h UTC on the date. Two types of the flight durations, short and long, were assumed because the true flight duration of the overseas migration is unknown.^36^ The short duration for which the backward trajectories end at dusk, 11.00 UTC on the previous date (terminal hour), ranges from 13 to 36 h, depending on the starting hour of 00.00–23.00 h, respectively. The long duration for which backward trajectories end at dusk two days before the date of interest ranges from 37 to 60 h, each expanding 24 h from the short duration.

##### Starting height

The flight height of *M. separata* observed by entomological radar in China were mostly between 200 and 1,000 m and sometimes up to 1,500 m at the end of May to mid-June.^50^^,^^51^ However, the flight height of overseas migration is unknown. This study, therefore, assumed 15 consecutive starting heights, from 100 to 1,500 m above ground level at 100-m intervals.

##### Flight speed

Noctuid moths, which are similar in size to *M. separata*, have a self-powered flight speed of 2.5–4.0 m/s^1^^,^^52^ The flight speed of *M. separata* ranges basically from 1.5 to 3.0 m/s, having a maximum of 4.0 m/s, in a flight mill experiment.^53^ In this trajectory calculation, the self-powered flight vector of 3.0 m/s at the downwind direction was assumed, so that the insect flies at 3.0 + wind speed (m/s) at its flight altitude.

##### Ambient air temperatures

Ambient air temperature affects the flight activity of *M. separata*.^53^ The maximum flight duration and flight distance in a flight mill experiment appeared at 11°C, and the values of both parameters decreased by 50% from the maximum at 9°C.^53^ The actogram showed that the flight activity was completely suppressed at temperatures below 3°C, was initiated at 4°C, and increased at between 5°C and 6°C, and then active flight activities appeared at temperatures of 7°C–8°C.^53^ In this modeling, it was assumed that *M. separata* flies for a long distance when the ambient air temperature at the flight height is ≥9°C. Therefore, a backward trajectory that went through cold upper air of <9°C became invalid and was removed. Backward trajectories with an ambient air temperature below 11 °C at terminal points (or take-off hours) were also excluded in this study because the emigrant insects were supposed to take off from the source at sufficiently high air temperatures.

##### Possible immigration dates

The time spent searching by each insect after its arrival in the destination and before its capture in the dry molasses or light traps was unknown. The three-day period before the date of sample collection was therefore considered a possible immigration period, because this temporal length was used in an analysis of overseas migration of the common cutworm, *Spodoptera litura*, searching for probable migration trajectories in the 3-day period.^35^ Since the trap-monitoring interval varied among monitoring sites and years (Table S2), the analytical period (possible immigration dates) also varied, from 3 days for the catch of daily monitoring to 9 days for the catch at 7-day monitoring intervals (Tables S2–S7). In the latter case, there were 9 possible immigration dates—i.e., the days of the 3-day period plus 6 non-survey days in the 7-day intervals.

### Quantification and statistical analyses

All statistical analysis and graphing were performed in R (version 4.0.5, https://www.r-project.org/) (accessed on 10th January 2024). The effect of locality in median of the Sr isotope ratios of the Japanese and Chinese references (hosts and insects) was tested with a Brunner-Munzel test (*brunnermunzel.test* of the package burunnermunzel in R). After confirming the difference, logistic regression (*glm*) was performed with the Chinese and Japanese reference data to examine the association of the origin of *M. separata* (the Chinese origin (1) or the origin of the Japanese immigration area (0); binary dependent variable) and the reference insect’s and host’s Sr radiogenic isotope ratio (scalar independent variable). The prediction model was evaluated with ROC (receiver operating characteristic) analysis (*roc* of the package pROC). An ^87^Sr/^86^Sr ratio cut-off that could be used to predict the origin of trapped insects was determined at the top left corner of the ROC curve (closest.topleft method). For discussion, the effect of locality in median of the Sr ratio of the wheat samples in the two Chinese provinces (Jiangsu and Henan) was similarly tested by Brunner-Munzel test.

## Supplemental information

Document S1. Figures S1–S10 and Tables S1–S8

Table S9. Sr isotopic data presented in the study, related to section “Data and code availability”

## References

[1] Drake V.A., Reynolds D.R. (2012).

[2] Jønsson K.A., Tøttrup A.P., Borregaard M.K., Keith S.A., Rahbek C., Thorup K. (2016). Tracking animal dispersal: from individual movement to community assembly. and global range dynamics. Trends Ecol. Evol..

[3] Schlägel U.E., Grimm V., Blaum N., Colangeli P., Dammhahn M., Eccard J.A., Hausmann S.L., Herde A., Hofer H., Joshi J. (2020). Movement-mediated community assembly and coexistence. Biol. Rev..

[4] Hu G., Lim K.S., Horvitz N., Clark S.J., Reynolds D.R., Sapir N., Chapman J.W. (2016). Mass seasonal bioflows of high-flying insect migrants. Science.

[5] Otuka A. (2013). Migration of rice planthoppers and their vectored re-emerging and novel rice viruses in East Asia. Front. Microbiol..

[6] Koyama J., Matsumura M. (2019). Ecology and control of armyworm, *Mythimna separata* (Lepidoptera: Noctuidae) in Japan, with special reference to outbreak and migration. Jpn. J. Appl. Entomol. Zool..

[7] Jiang X., Luo L., Zhang L., Sappington T.W., Hu Y. (2011). Regulation of migration in *Mythimna separata* (Walker) in China: a review integrating environmental, physiological, hormonal, genetic, and molecular factors. Environ. Entomol..

[8] Hirai K., Mita H. (1983). Comparative physio-ecological studies on the armyworm, *Pseudaletia separata* Walker and *Leucania loreyi* Duponchel (Lepidoptera: Noctuidae). Bull. Chugoku Agric. Exp. Stn..

[9] Hirai K. (1990). Relationship of weather in overwintering areas to outbreaks of the armyworm, *Pseudaletia separata* WALKER (Lepidoptera: Noctuidae). Jpn. J. Appl. Entomol. Zool..

[10] Lee J.-H., Uhm K.-B., Drake V.A., Gatehouse A.G. (1995). Insect migration: tracking resources through space and time.

[11] Li K.-P., Wong H.-H., Woo W.-S. (1964). Route of the seasonal migration of the oriental armyworm moth in the eastern part of China as indicated by a three-year result of releasing and recapturing of marked moths. Acta Phytophylacica Sin..

[12] Jiang Y.-Y., Liu J., Zeng J. (2018). Using a national searchlight trap network to monitoring the annual dynamics of the oriental armyworm in China. Chinese J. Appl. Entomol..

[13] Feng H.Q., Zhao X.C., Wu X.F., Wu B., Wu K.M., Cheng D.F., Guo Y.Y. (2008). Autumn migration of *Mythimna separata* (Lepidoptera: Noctuidae) over the Bohai Sea in northern China. Environ. Entomol..

[14] Cang X., Zhao S., Yang X., Yuan H., Liu J., Liu D., Yang X., Wu K. (2023). Migration monitoring and route analysis of the oriental armyworm *Mythimna separata* (Walker) in Northeast China. Agronomy.

[15] Tong D., Zhang L., Wu N., Xie D., Fang G., Coates B.S., Sappington T.W., Liu Y., Cheng Y., Xia J. (2022). The oriental armyworm genome yields insights into the long-distance migration of noctuid moths. Cell Rep..

[16] Hsia T.-S., Tsai S.-M., Ten H.-S. (1963). Studies of the regularity of outbreak of the oriental armyworm, *Leucania separata* Walker. Acta Entomol. Sin..

[17] Oku T., Kobayashi T. (1974). Early summer outbreaks of the oriental armyworm, *Mythimna separata* Walker, in the Tohoku district and possible causative factors (Lepidoptera: Noctuidae). Appl. Entomol. Zool..

[18] Hobson K.A., Wassenaar L.I., Taylor O.R. (1999). Stable isotopes (δD and δ^13^C) are geographic indicators of natal origins of monarch butterflies in eastern North America. Oecologia.

[19] Borisov S.N., Iakovlev I.K., Borisov A.S., Ganin M.Y., Tiunov A.V. (2020). Seasonal Migrations of *Pantala flavescens* (Odonata: Libellulidae) in Middle Asia and Understanding of the Migration Model in the Afro-Asian Region Using Stable Isotopes of Hydrogen. Insects.

[20] Hobson K.A., Barnett-Johnson R., Cerling T., West J.B., Bowen G.J., Dawson T.E., T K.P. (2010). Isoscape: Understanding Movement, Pattern, and Process.

[21] Quinby B.M., Creighton J.C., Flaherty E.A. (2020). Stable isotope ecology in insects: a review. Ecol. Entomol..

[22] Bowen G.J. (2010). Isoscapes: Spatial Pattern in Isotopic Biogeochemistry. Annu. Rev. Earth Planet Sci..

[23] Crowley B.E., Miller J.H., Bataille C.P. (2017). Strontium isotopes (^87^Sr/^86^Sr) in terrestrial ecological and palaeoecological research: empirical efforts and recent advances in continental-scale models. Biol. Rev..

[24] Bataille C.P., Crowley B.E., Wooller M.J., Bowen G.J. (2020). Advances in global bioavailable strontium isoscapes. Palaeogeogr. Palaeoclimatol. Palaeoecol..

[25] Agriculture F., Fisheries Research Council Secretariat (AFFRCS) (1989).

[26] Aoyama K., Nakano T., Shin K.C., Izawa A., Morita S. (2017). Variation of strontium stable isotope ratios and origins of strontium in Japanese vegetables and comparison with Chinese vegetables. Food Chem..

[27] Xu Z., Li Y., Tang Y., Han G. (2009). Chemical and strontium isotope characterization of rainwater at an urban site in Loess Plateau, Northwest China. Atmos. Res..

[28] Alonzi E., Pacheco-Forés S.I., Gordon G.W., Kuijt I., Knudson K.J. (2020). New understandings of the sea spray effect and its impact on bioavailable radiogenic strontium isotope ratios in coastal environments. J. Archaeol. Sci. Rep.

[29] Holder P.W., Armstrong K., Van Hale R., Millet M.-A., Frew R., Clough T.J., Baker J.A. (2014). Isotopes and trace elements as natal origin markers of *Helicoverpa armigera* - an experimental model for biosecurity pests. PLoS One.

[30] Reich M.S., Flockhart D.T.T., Norris D.R., Hu L., Bataille C.P. (2021). Continuous-surface geographic assignment of migratory animals using strontium isotopes: A case study with monarch butterflies. Methods Ecol. Evol..

[31] Teraoka Y., Okumura K. (2011).

[32] Talavera G., Bataille C., Benyamini D., Gascoigne-Pees M., Vila R. (2018). Round-trip across the Sahara: Afrotropical Painted Lady butterflies recolonize the Mediterranean in early spring. Biol. Lett..

[33] Jia H., Liu Y., Li X., Li H., Pan Y., Hu C., Zhou X., Wyckhuys K.A.G., Wu K. (2022). Windborne migration amplifies insect-mediated pollination services. Elife.

[34] Wu M.F., Qi G.J., Chen H., Ma J., Liu J., Jiang Y.Y., Lee G.S., Otuka A., Hu G. (2022). Overseas immigration of the fall armyworm, *Spodoptera frugiperda* (Lepidoptera: Noctuidae), invading Korea and Japan in 2019. Insect Sci..

[35] Tojo S., Ryuda M., Fukuda T., Matsunaga T., Choi D.R., Otuka A. (2013). Overseas migration of the common cutworm, *Spodoptera litura* (Lepidoptera: Noctuidae). Appl. Entomol. Zool..

[36] Otuka A., Niiyama T., Jiang X.-F. (2023). Possible source and migration pathway for early-summer immigrants of the oriental armyworm, *Mythimna separata*, arriving in northern Japan. J. Integr. Agric..

[37] Yamamoto M., Kojima T., Hasegawa Y., Doi S. (1993). Studies on the seasonal abundance of the armyworm, *Mythimna separata* Walker, in Shiga prefecture by using molasses bait traps and synthetic sex pheromone traps. Bull. Shiga Pref. Agric. Exp. Stn..

[38] Tanaka A. (1976). The occurrence of oriental armyworm, *Mythimna separata*, - topics centred on the normal occurrence of moth-. Plant Prot..

[39] Otuka A., Matsumura M., Sanada-Morimura S., Takeuchi H., Watanabe T., Ohtsu R., Inoue H. (2010). The 2008 overseas mass migration of the small brown planthopper, *Laodelphax striatellus*, and subsequent outbreak of rice strip disease in western Japan. Appl. Entomol. Zool..

[40] Liu H., Wei Y., Lu H., Wei S., Jiang T., Zhang Y., Guo B. (2016). Combination of the ^87^Sr/^86^ Sr ratio and light stable isotopic values (δ^13^ C, δ^15^ N and δD) for identifying the geographical origin of winter wheat in China. Food Chem..

[41] Liu H., Wei Y., Lu H., Wei S., Jiang T., Zhang Y., Ban J., Guo B. (2017). The determination and application of^87^ Sr/^86^ Sr ratio in verifying the geographical origin of wheat. J. Mass Spectrom..

[42] Frei R., Frei K.M., Jessen S. (2020). Shallow retardation of the strontium isotope signal of agricultural liming - implications for isoscapes used in provenance studies. Sci. Total Environ..

[43] Asahina S., Tsuruoka Y. (1967). Records of the insects visited a weather ship located at the Ocean Weather Station "Tango" on the Pacific. Kontyu.

[44] Kiritani K., Hirai Y. (1987). Japan-China cooperative study on the long-range migration of the white-backed planthopper. Plant Prot..

[45] Otuka A., Nagayoshi K., Sanada-Morimura S., Matsumura M., Haraguchi D., Kakazu R. (2016). Estimation of possible sources for wind borne re-invasion of *Bactrocera dorsalis* complex into islands of Okinawa Prefecture, southwestern Japan. Appl. Entomol. Zool..

[46] Riley J.R., Reynolds D.R., Smith A.D., Edwards A.S., Zhang X.-X., Cheng X.-N., Wang H.-K., Cheng J.-Y., Zhai B.-P. (1995). Observations of the autumn migration of the rice leaf roller *Cnaphalocrocis medinalis* (Lepidoptera: Pyralidae) and other moths in eastern China. Bull. Entomol. Res..

[47] Close R.C., Moar N.T., Tomlinson A.I., Lowe A.D. (1978). Aerial dispersal of biological material from Australia to New Zealand. Int. J. Biometeorol..

[48] Faure G. (2001).

[49] Draxler R.R., Hess G.D. (1997). NOAA Tech. Memo. ERL ARL-224.

[50] Chen R.-L., Bao X.-Z., Drake V.A., Farrow R.A., Wang S.-Y., Sun Y.-J., Zhai B.-P. (1989). Radar observations of the spring migration into northeastern China of the oriental armyworm, *Mythimna separata*, and other insects. Ecol. Entomol..

[51] Chen R.L., Sun Y.J., Wang S.Y., Zhai B.P., Bao X.Z., Drake V.A., Gatehouse A.G. (1995). Insect Migration: Tracking Resource in Space and Time.

[52] Minter M., Pearson A., Lim K.S., Wilson K., Chapman J.W., Jones C.M. (2018). The tethered flight technique as a tool for studying life-history strategies associated with migration in insects. Ecol. Entomol..

[53] Zhang Z., Li G. (1985). A study the biological characteristics of the flight of the oriental armyworm [*Mythimna separata* (Walker)] moth. Phytophylacica Sinca.

