# Supplementary material for: Strontium isotope and trajectory method elucidating overseas migration of Mythimna separata to Japan
Source: iScience. 2024 Oct 11;27(11):111160. doi: 10.1016/j.isci.2024.111160 (PMC11544078; doi:10.1016/j.isci.2024.111160)
Supplement: Document S1. Figures S1–S10 and Tables S1–S8 [file mmc1.pdf]

iScience, Volume *27*

Supplemental information

Strontium isotope and trajectory method elucidating overseas migration of *Mythimna separata* to Japan

Naoya Hidaka, Caihong Tian, Shengnan Zhang, Gaku Akiduki, Guoping Li, Ichiro Tayasu, Ki-Cheol Shin, Tokumitsu Niiyama, Gao Hu, Shimin Li, Akira Otuka, and Hongqiang Feng

This supplementary information includes supplementary figures, tables and codes.

Supplementary figures

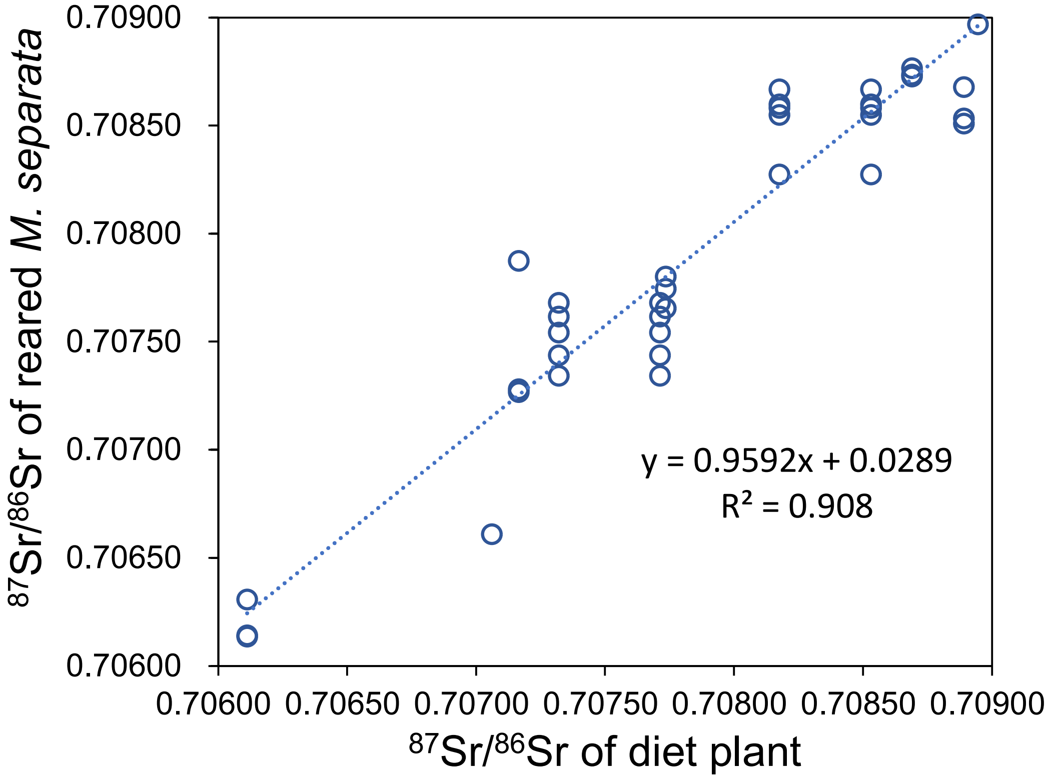

Figure S1. Linear relationship between the $^{87}Sr/^{86}Sr$ ratios of diet plants and reared *M. separata* in the Japanese breeding experiment, related to section "Strontium isotope ratio of the reference"

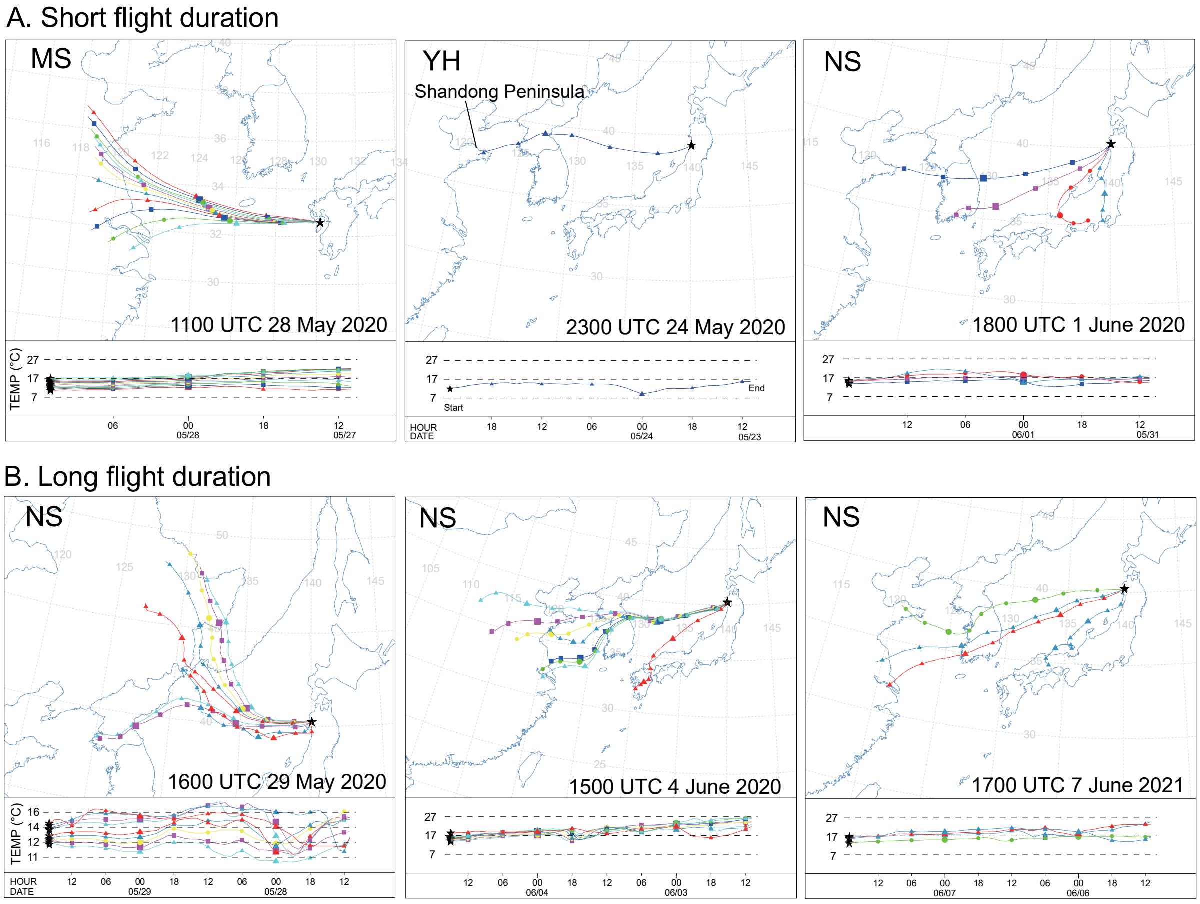

Figure S2. Typical *M. separata* backward trajectories, related to section "Trajectory analysis"

(A) Short flight duration (min 13 h, max 36 h). (B) Long flight duration (min 37 h, max 60 h). The trajectories reached over the land on the upper map and their ambient air temperature (°C) along the trajectories in the lower graph.

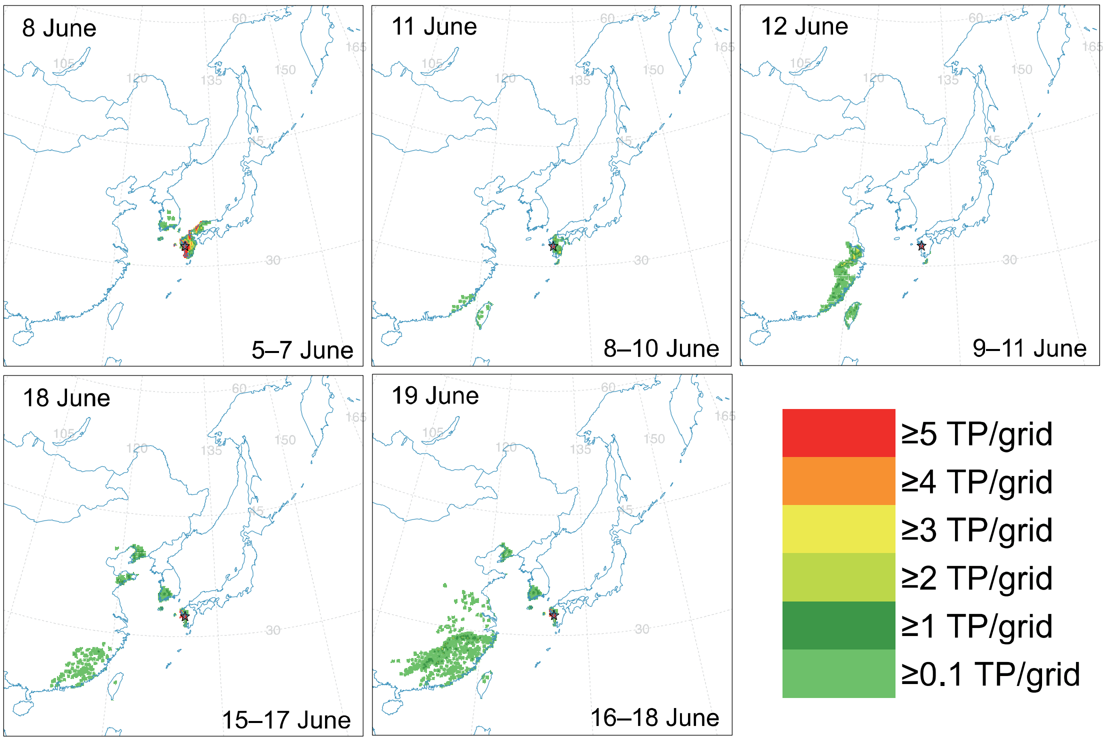

Figure S3. Frequency map of the terminal point of backward trajectories with short duration (min 13 h, max 36 h) starting at MS in Nagasaki prefecture in 2020, related to section "Trajectory analysis"

The maps of dates when $^{87}Sr/^{86}Sr$ ratio of the insect catches is below the cut-off $^{87}Sr/^{86}Sr$ ratio are shown. The date of sample collection is shown in the upper line, and the analytical period in the lower line in each panel.

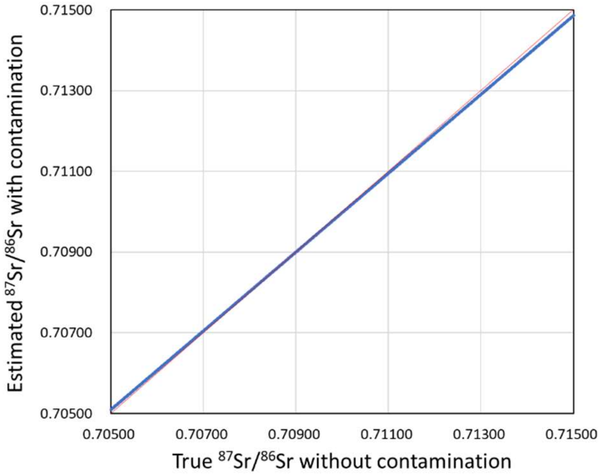

Figure S4. Contamination level of the Japanese measurement, related to STAR Methods.

The *blue line* and *red line* indicate estimated $^{87}Sr/^{86}Sr$ ratio of a model sample containing Sr of 7.278 ng with contamination of 0.175 ng and a linear line, respectively. The contamination's $^{87}Sr/^{86}Sr$ ratio of 0.70905 was assumed.

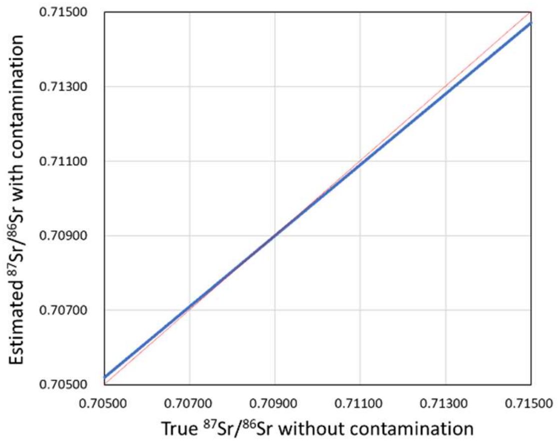

Figure S5. Contamination level of the Chinese measurement, related to STAR Methods.

The *blue line* and *red line* indicate estimated $^{87}Sr/^{86}Sr$ ratio of a model sample containing Sr of 7.278 ng with a contamination of 0.37 ng and a linear line, respectively. The contamination's $^{87}Sr/^{86}Sr$ ratio of 0.70905 was assumed like the Japanese estimation above.

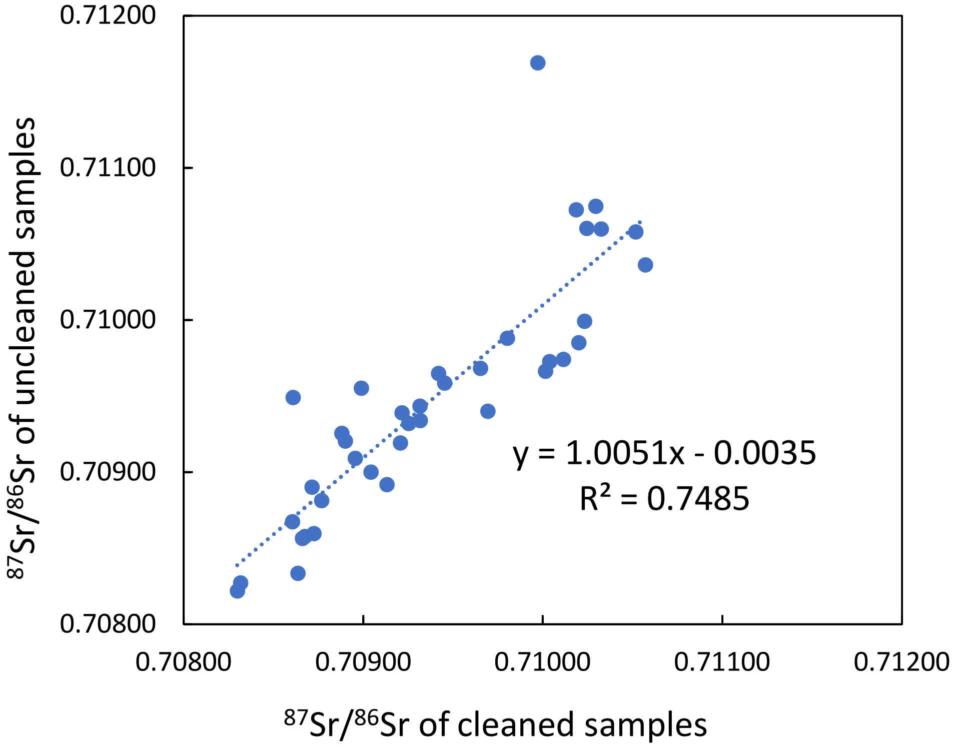

Figure S6. Comparison between the $^{87}Sr/^{86}Sr$ ratios of insects trapped at MS and NS with and without nitrogen-gas cleaning, related to STAR Methods

Each adult sample without abdomen and forewings was cut in two along the central body axis. One half of the dissected body was cleaned twice with a pure nitrogen gas flow of 350 kPa for 30 seconds each time before acid digestion in the laboratory, and the other half was not cleaned. The linearity (the blue dotted line) was found between the two groups (Table S9).

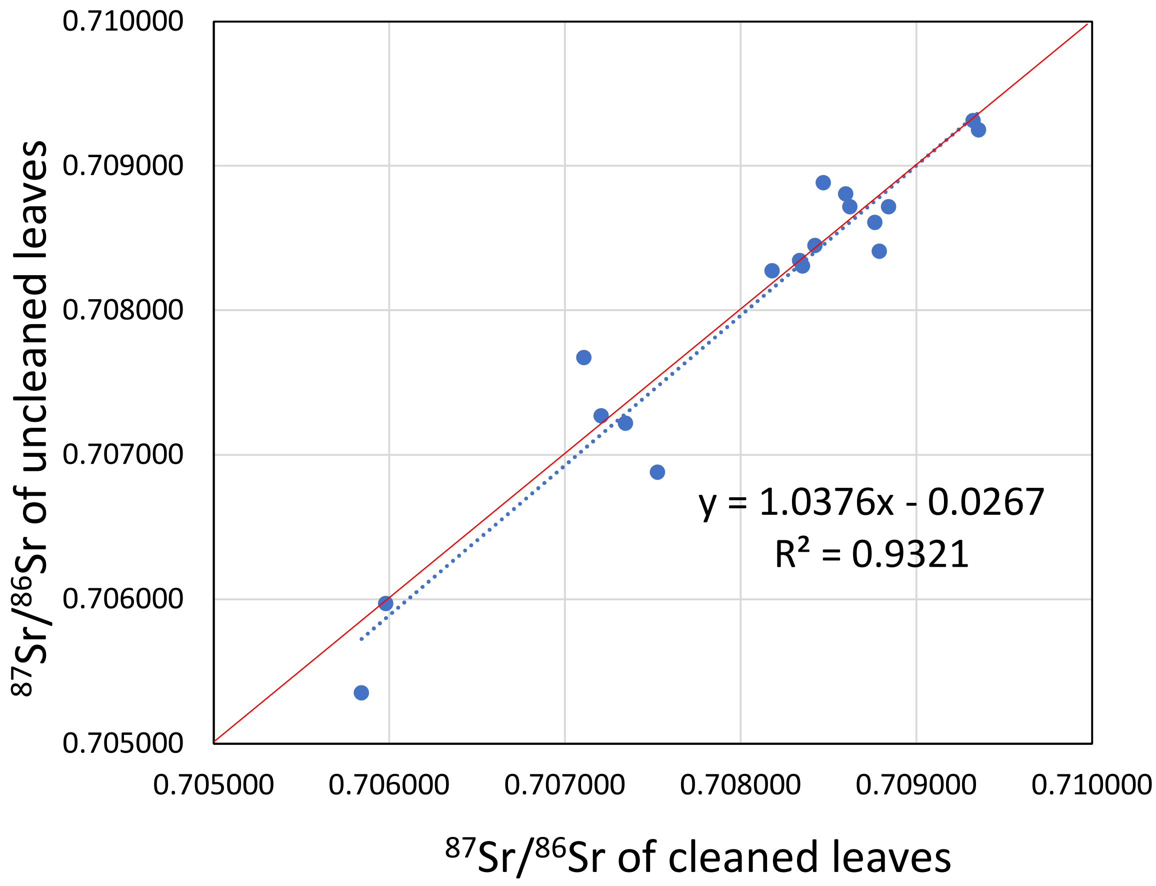

Figure S7. Linearity between the $^{87}Sr/^{86}Sr$ ratio of cleaned and uncleaned leaves of Solanaceae plants, related to STAR Methods

The *blue dotted line* and *red line* indicate a linear regression line and the linear line, respectively (Table S9). As reference, the effect of ultrasonic cleaning of Solanaceae plant leaves was tested. The plants were collected from farmers' fields in Kyushu district, the same as in this study (Table S9). Each plant in a clean plastic bag were stored in a freezer before the test. A part of a leaf or leaves were cut with a ceramic scissors in the laboratory condition and dipped into ultra clean water in a clean beaker, to which ultrasound (35 kHz, 110 W) for 8 min was applied for 3 times in total（Ultrasonic cleaner, AS 486，AS ONE Cooperation, Osaka).

The $^{87}Sr/^{86}Sr$ ratios of cleaned and uncleaned leaves indicated a good linearity between the two groups (Figure S7).

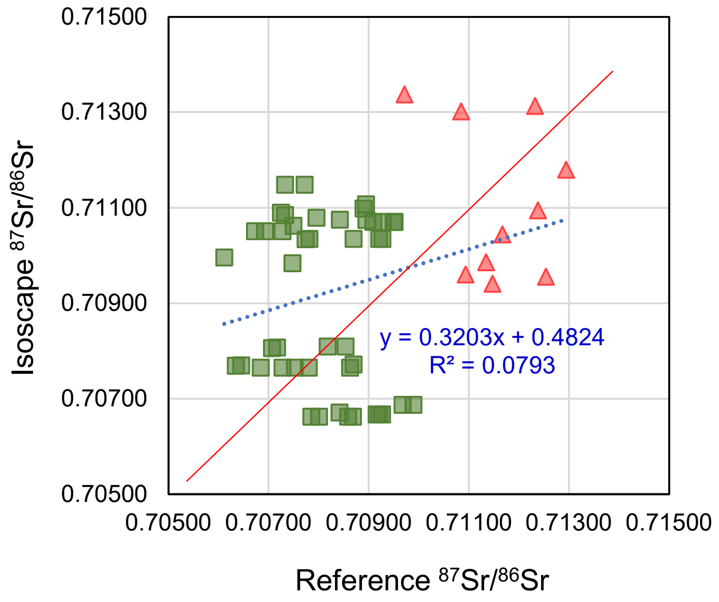

Figure S8. Relationship between the $^{87}Sr/^{86}Sr$ ratio of plant samples in East Asian agricultural fields and that of the isoscape by Bataille et al. [S1], related to section "The $^{87}Sr/^{86}Sr$ ratio of agricultural soils"

The *green square* and *red triangle* indicate Japanese and Chinese samples, respectively. The *blue dotted line* and *red line* indicate a linear regression line and a linear line, respectively. See the data list (Table S9).

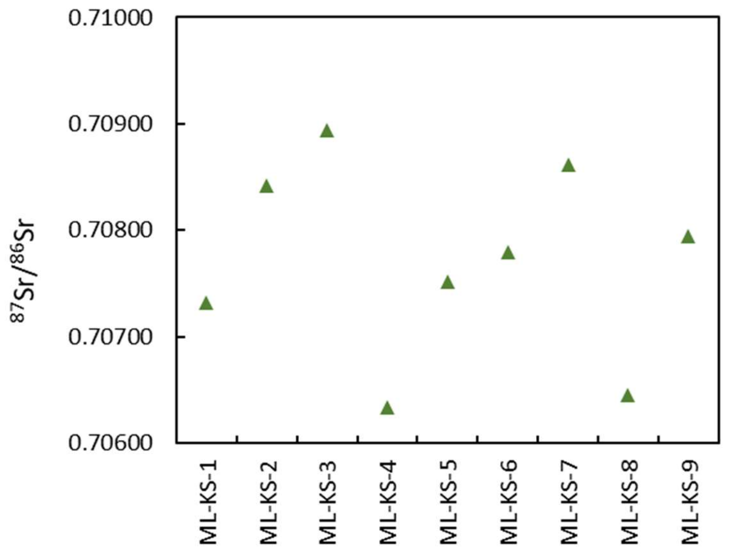

Figure S9. The $^{87}Sr/^{86}Sr$ ratios of leaves of feed maize collected in Koshi city, related to section "The $^{87}Sr/^{86}Sr$ ratio of agricultural soils"

ML-KS-*n* is the leaf sample ID in Table S8.

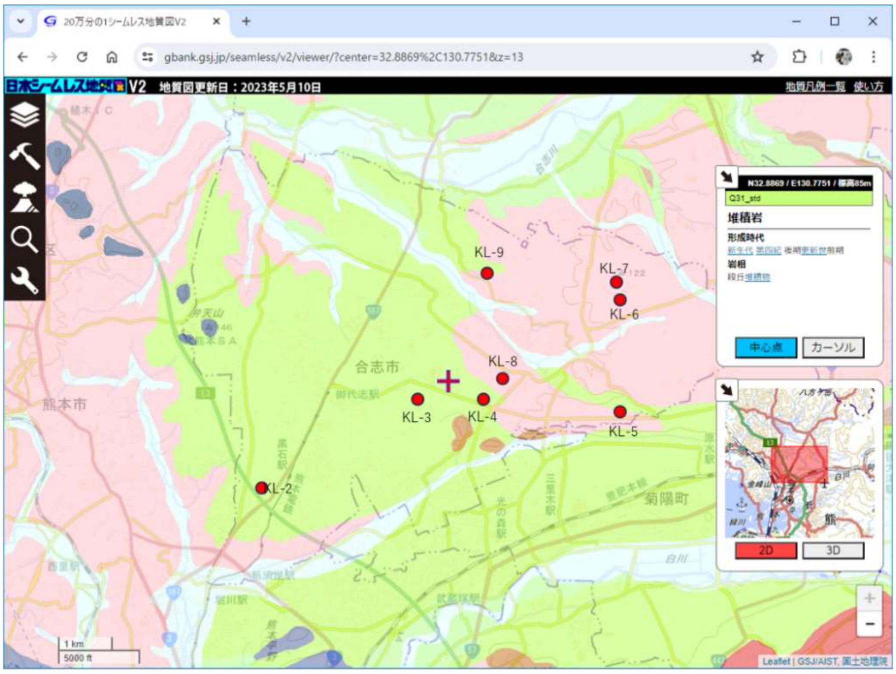

Figure S10. Geological map of the sampling points in Koshi city (the Geological Survey of Japan: https://gbank.gsj.jp/seamless/), related to Figure S9
The *red circle* indicates a sampling point with Field ID (Table S8).

Supplementary tables

Table S1. Collection sites of host plants and *M.separata* in Japan and China, related to STAR Methods

Code	City/Town	Prefecture/ Province	Country	Latitude (°N)	Longitude (°E)
GG	Goshogawara	Aomori	Japan	41.05	140.36
SH	Shichinohe	Aomori	Japan	40.64	141.14
NS	Noshiro	Akita	Japan	40.24	140.05
KU	Kotoura	Tottori	Japan	35.48	133.70
CG	Chikugo	Fukuoka	Japan	33.21	130.49
KS	Koshi	Kumamoto	Japan	32.91	130.78
UZ	Unzen	Nagasaki	Japan	32.88	130.29
TH	Takaharu	Miyazaki	Japan	31.93	130.99
MJ	Miyakonojyo	Miyazaki	Japan	31.77	131.14
MN	Minami-Satsuma	Kagoshima	Japan	31.48	130.34
KT	Kimotsuki	Kagoshima	Japan	31.33	130.95
LYG	Lianyungang	Jiangsu	China	34.33	119.29
LYG	Lianyungang	Jiangsu	China	34.42	119.51
YC	Yancheng	Jiangsu	China	33.78	119.81
TZ	Taizhou	Jiangsu	China	32.51	120.13
NT	Nantong	Jiangsu	China	32.11	121.08
XX	Xinxiang	Henan	China	35.00	113.70
LY	Luoyang	Henan	China	34.66	112.48
LH	Luohe	Henan	China	33.62	113.98
NY	Nanyang	Henan	China	32.75	113.00
XY	Xinyang	Henan	China	32.03	114.27

Table S2. Trap monitoring data of *M. separata* in Akita and Nagasaki prefectures, Related to section “Trajectory analysis”

Date	Trap catch			Date	Trap catch
	MS	YH	NS		NS
5/20/2020	0			5/20/2021	0
5/21/2020	2		0	5/21/2021	0
5/22/2020	0			5/22/2021	
5/23/2020				5/23/2021	
5/24/2020				5/24/2021	
5/25/2020	2			5/25/2021	
5/26/2020	6	1	1	5/26/2021	
5/27/2020	6	0		5/27/2021	1
5/28/2020	2	1		5/28/2021	
5/29/2020	29		0	5/29/2021	
5/30/2020		0		5/30/2021	
5/31/2020			1	5/31/2021	
6/1/2020	41*			6/1/2021	
6/2/2020	15	0		6/2/2021	1
6/3/2020	11	0		6/3/2021	
6/4/2020	18		0	6/4/2021	
6/5/2020	7			6/5/2021	
6/6/2020				6/6/2021	
6/7/2020				6/7/2021	
6/8/2020	15	0	4	6/8/2021	
6/9/2020	5			6/9/2021	
6/10/2020	4		4	6/10/2021	
6/11/2020	2	0		6/11/2021	
6/12/2020	2		1	6/12/2021	
6/13/2020		0		6/13/2021	
6/14/2020				6/14/2021	
6/15/2020	1			6/15/2021	1
6/16/2020	0	0	0	6/16/2021	
6/17/2020	1			6/17/2021	0
6/18/2020	3			6/18/2021	
6/19/2020	1			6/19/2021	
6/20/2020				6/20/2021	

Empty cell indicates no survey was conducted.
* Samples on 1 June 2020 at MS were unavailable due to loss.

Table S3. Monitoring parameters, related to STAR Methods

Code	Locality	Prefecture	Trap type	Latitude (°N)	Longitude (°E)	Start date	End date
YH	Yurihonjo	Akita	Dry molasses trap	39.28	140.08	21/04/2020	16/06/2020
NS	Noshiro	Akita	Dry molasses trap	40.24	140.05	16/04/2020	16/06/2020
NS	Noshiro	Akita	Dry molasses trap	40.24	140.05	28/04/2021	17/06/2021
MS	Minami-Shimabara	Nagasaki	Searchlight trap	32.60	130.18	25/03/2020	19/06/2020

Table S4. Sr concentration of the blanks before column Sr separation, related to STAR Methods

ID	Total Sr (ng)
Blank 1	0.170
Blank 2	0.165
Blank 3	0.221
Blank 4	0.169
Blank 5	0.151
Average	0.175
Stdev	0.027

Blank-1,2 started at sample preparation in the laboratory of NARO, and blank-3,4,5 started at first digestion in the cleanroom of RIHN. The Sr concentration was measured with Agilent ICP-MS 8900 (ICP-QQQ).

Table S5. Sr concentration of the blanks after column Sr separation, related to STAR Methods

ID	Total Sr (ng) remaining
Blank 1	0.060
Blank 2	0.057
Blank 3	0.066
Blank 4	0.044
Blank 5	0.045
Sum	0.272

The Sr concentration was measured with Agilent ICP-MS 8900 (ICP-QQQ).

Table S6. Chinese elution method, related to STAR Methods

Eluents	Volume (mL), Repetition (times)	Elution
Milli-Q Water	2, 10	Resin cleaning
8 N HNO_3	1, 5	Column preconditioning
Sample solution	1, 5	Sample introduction
8 N HNO_3	2, 4	Wash matrix
0.05 N HNO_3	2, 4	Elute Sr for wheat leaves
0.1 N HNO_3	4, 5	Elute Sr for *M. separata*

Table S7. Analytical period to calculate backward trajectories for trap catch, Related to STAR Methods

Locality	Date of sample collection (UTC) *	Analytical period (UTC) **	Days of the period
YH	26 May 2020	17 - 25 May 2020	9
YH	28 May 2020	24 - 27 May 2020	4
NS	31 May 2020	26 - 30 May 2020	5
NS	8 June 2020	1 - 7 June 2020	7
NS	10 June 2020	7 - 9 June 2020	3
NS	27 May 2021	18 - 26 May 2021	9
NS	15 June 2021	30 May - 14 June 2021	16
MS	21 May 2020	18 - 20 May 2020	3
MS	25 May 2020	20 - 24 May 2020	5
MS	27 May 2020	24 - 26 May 2020	3
MS	28 May 2020	25 - 27 May 2020	3

MS	29 May 2020	26 - 28 May 2020	3
MS	2 June 2020	30 May - 1 June 2020	3
MS	3 June 2020	31 May - 2 June 2020	3
MS	4 June 2020	1 - 3 June 2020	3
MS	5 June 2020	2 - 4 June 2020	3
MS	8 June 2020	3 - 7 June 2020	5
MS	9 June 2020	6 - 8 June 2020	3
MS	10 June 2020	7 - 9 June 2020	3
MS	11 June 2020	8 - 10 June 2020	3
MS	12 June 2020	9 - 11 June 2020	3
MS	18 June 2020	15 - 17 June 2020	3
MS	19 June 2020	16 - 18 June 2020	3

* Sample collection was conducted in the morning, or from 0000 to 0300 h UTC. Catch number is shown in Table S2.

** Length of the analytical period was set to 3 days for the catch of daily monitoring, and became longer (4 - 9 days) for the catch at 2-day to 7-day monitoring intervals, respectively (Table S2)

Table S8. Sampling parameters for the maize leaves collected in Koshi city, Related to Figure S9

Sample ID	Plant	City	Field ID	Latitude(°N)	Longitude (°E)
ML-KS-1	Maize for feed	Koshi, Kumamoto	KL-2	32.87	130.73
ML-KS-2	Maize for feed	Koshi, Kumamoto	KL-3	32.88	130.77
ML-KS-3	Maize for feed	Koshi, Kumamoto	KL-4	32.88	130.78
ML-KS-4	Maize for feed	Koshi, Kumamoto	KL-5	32.88	130.81
ML-KS-5	Maize for feed	Koshi, Kumamoto	KL-6	32.90	130.81
ML-KS-6	Maize for feed	Koshi, Kumamoto	KL-6	32.90	130.81
ML-KS-7	Maize for feed	Koshi, Kumamoto	KL-7	32.90	130.81
ML-KS-8	Maize for feed	Koshi, Kumamoto	KL-8	32.88	130.79
ML-KS-9	Maize for feed	Koshi, Kumamoto	KL-9	32.90	130.78

Program codes

Programs to make frequency Figures 3, 4 and S3 were listed. They are related to STAR Methods. Contact the lead contact on how to use these programs.

Program 1:

This shell program repeatedly calculates a single trajectory from a specified location (trap site) with different starting heights and times for a single date.

```
#!/bin/csh
if( $#argv < 3 ) then
        echo “usage: gohysplit.sh yy mm dd”
        exit
endif

set place=noshiro
set slat=40.24
set slong=140.05
set year=$1

cd /Users/aotuka/hysplit/working
foreach height ( 1500 1400 1300 1200 1100 1000 900 800 700 600 500 400 300 200 100 )

  set hs=23
```

```
  while($hs > -1)
        set he=`echo "($hs+13)*(-1)"|bc`
        echo $1 $2 $3 $hs >CONTROL
        echo 1 >>CONTROL
        echo $slat $slong $height >>CONTROL
        echo $he >>CONTROL
        echo 1 >>CONTROL
        echo 10000.0 >>CONTROL
        echo 10 >>CONTROL
        echo /Users/aotuka/hysplit/working/ >>CONTROL
        echo gdas1.may$year.w1 >>CONTROL
        echo /Users/aotuka/hysplit/working/ >>CONTROL
        echo gdas1.may$year.w2 >>CONTROL
        echo /Users/aotuka/hysplit/working/ >>CONTROL
        echo gdas1.may$year.w3 >>CONTROL
        echo /Users/aotuka/hysplit/working/ >>CONTROL
        echo gdas1.may$year.w4 >>CONTROL
        echo /Users/aotuka/hysplit/working/ >>CONTROL
        echo gdas1.may$year.w5 >>CONTROL
        echo /Users/aotuka/hysplit/working/ >>CONTROL
        echo gdas1.jun$year.w1 >>CONTROL
        echo /Users/aotuka/hysplit/working/ >>CONTROL
        echo gdas1.jun$year.w2 >>CONTROL
        echo /Users/aotuka/hysplit/working/ >>CONTROL
        echo gdas1.jun$year.w3 >>CONTROL
        echo /Users/aotuka/hysplit/working/ >>CONTROL
        echo gdas1.jun$year.w4 >>CONTROL
        echo /Users/aotuka/hysplit/working/ >>CONTROL
        echo gdas1.jun$year.w5 >>CONTROL
        echo ./ >>CONTROL
        echo tdump>>CONTROL

        /Users/aotuka/hysplit/exec/hyts_std
        if($hs > 22) then
                mkdir $place
                mkdir $place/$1.freq
        endif
        cp tdump $place/$1.freq/tdump.$1$2$3.$hs.$height
          echo $height $hs
        set hs=`echo "$hs-1"|bc`
  end # while
end # foreach
```

Program 2:

This shell program removes an invalid trajectory from a trajectory list by a program exep_tempf_9_11dgrC, counts frequency of terminal point of valid trajectories in each grid cell of 0.25 by 0.25 degree by trajfreq, and make a frequency map by concplot.

```
#!/bin/csh
ls tdump.* >./list
foreach fname (`cat ./list`)
  exep_tempf_9_11dgrC ./$fname `cat ./$fname|wc -l` >./ep_$fname
end
ls ep_* >./eplist
```

```
../../../exec/trajfreq –feplist.bin –g0.25 –i./eplist –a0 –r0 –s0:1500
../../../exec/concplot –i./eplist.bin –j../../../graphics/arlmap –k2 –h40.0:130.0 –g0:1500 –x$1 –v5+4+3+2+1+0.1 +m0 –z100
convert –density 200 –background white –flatten ./concplot.ps ./concplot.pdf
```

Program 3:

This program is C source code of exep_tempf_9_11dgrC that is used by Program 2. It removes nodes of the invalid trajectory based on ambient temperatures at the nodes.

```
#include <stdio.h>
#include <stdlib.h>
#include <string.h>
#define N 128
#define TMIN 282.15
#define TTAKEOFF 284.15
#define NHEADER 15

int main(int argc, char *argv[])
{
	FILE *fp;
	char fname[128];
	char line[N],cpline[N];
	int counter=1;
	float tamb;
	int invalid=0;
	int itakeoff=0;
	strcpy(fname, argv[1]);

	fp = fopen(fname, “r”);
	if(fp == NULL) {
		printf(“%s file not open!¥n”, fname);
		return -1;
	}

	while(fgets(line, N, fp) != NULL) {
		strcpy(cpline, line);
		if (counter <= NHEADER)
			printf(“%s”, line);
		if (counter >= NHEADER)
		{
			tamb = atof(&line[95]);
			if(tamb <= TMIN)
				invalid += 1;
			if(tamb > TTAKEOFF){
				itakeoff = 1;
			}else{
				itakeoff = 0;
			}
		}
		counter++;
	}
	if (invalid == 0 && itakeoff == 1) printf(“%s”,cpline);
```

```
    fclose(fp);

    return 0;
}
```

Reference

S1. Bataille, C.P., Crowley, B.E., Wooller, M.J., Bowen, G.J. (2020). Advances in global bioavailable strontium isoscapes. Palaeogeogr. Palaeoclimatol. Palaeoecol. 555, 109849. https://doi.org/10.1016/j.palaeo.2020.109849
